# Estimating cross-border cloud computing emissions: A consumption-based approach applied to major European data center hubs

**DOI:** 10.1016/j.isci.2026.116061

**Published:** 2026-05-22

**Authors:** Ian V. Soares, Anna Furberg, Shoaib Azizi, Göran Finnveden, Masaru Yarime, Magdalena M. Klemun

**Affiliations:** 1 Division of Public Policy (PPOL) - The Hong Kong University of Science and Technology (HKUST), Clearwater Bay, Kowloon, Hong Kong; 2 KTH Digital Futures, KTH Climate Action Center, Department of Sustainable Development, Environmental Science and Engineering, KTH Royal Institute of Technology, 100 44 Stockholm, Sweden; 3 Whiting School of Engineering, Department of Civil and Systems Engineering - John Hopkins University, 3400 North Charles Street, Baltimore, MD, USA

**Keywords:** computing, energy sustainability, energy systems

## Abstract

Cloud computing’s expansion creates a significant carbon footprint from data center energy consumption. Standard location-based accounting attributes cloud emissions to countries where data centers are located, potentially obscuring where actual demand lies when services are consumed across borders. This study develops a consumption-based accounting approach to estimate cross-border cloud emission flows among the EU’s three largest data center hubs: Germany, Ireland, and the Netherlands, from 2017 to 2022. Using electricity consumption and emissions for data centers and telecommunications networks, we classify cloud activity as domestic (hosted and consumed within one country), export (domestic hosting, foreign consumption), or import (foreign hosting, domestic consumption). The analysis reveals distinct profiles: Germany’s cloud footprint is predominantly domestic (75%), Ireland’s is export-dominated (85% serves foreign consumers), and the Netherlands exhibits balanced flows. Consumption-based metrics can complement territorial accounting by revealing cross-border emission flows relevant to national climate planning and infrastructure policy decisions.

## Highlights

• Location-based accounting allocated cloud emissions only to host countries
• The consumption approach reveals that countries have distinct cloud use profiles
• Shifting cloud emissions to low-carbon grids lowers aggregate cloud emissions
• Using consumption with location accounting improves the accuracy of cloud emissions

## Graphical abstract

**Figure:**
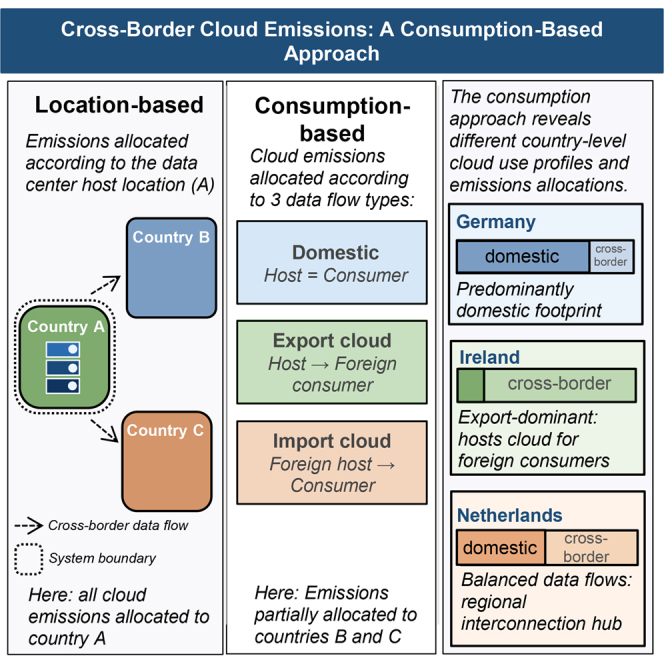

## Introduction

Research on information and communication technology (ICT) emissions has traditionally focused on location-based accounting, which attributes emissions to the countries where data centers (DCs) and telecommunications networks (TNs) are physically located.^1^^,^^2^^,^^3^^,^^4^ While this method aligns with conventional greenhouse gas inventory practices, several studies highlight that cloud computing services are inherently transboundary, as demand and infrastructure are often geographically decoupled.^2^^,^^5^^,^^6^ This spatial separation can lead to a divergence between where emissions and local environmental impacts are recorded and where digital services are consumed, raising questions about how responsibility is attributed.

These challenges mirror a well-established debate in climate policy between territorial and consumption-based accounting.^7^^,^^8^ Territorial accounting attributes emissions to the locations where they occur, forming the basis for national reporting and international climate negotiations. In contrast, consumption-based accounting allocates emissions to the end users of goods or services, offering a complementary lens that captures emissions embodied in trade and cross-border flows. This perspective has gained traction for assessing global supply chains and traded goods,^9^ yet its application to digital infrastructure remains limited. As digitalization accelerates, extending this framework to cloud computing is increasingly important for understanding the actual geography of carbon responsibility.

Previous work has quantified emissions from DCs and networks at regional and global levels, improving estimates of ICT’s overall energy use and carbon footprint.^5^^,^^10^^,^^11^^,^^12^ Related studies have examined European electricity and emissions trends,^13^^,^^14^^,^^15^^,^^16^^,^^17^ the energy intensity of data transmission,^18^^,^^19^^,^^20^ and cross-border digital flows.^21^ Collectively, these studies have advanced current understanding of ICT’s energy footprint. However, a critical gap remains: existing research has not applied consumption-based accounting principles to cloud computing, leaving the transboundary dimension of digital emissions largely uninvestigated. This gap is consequential because cloud services involve minimal physical transport yet generate substantial cross-border electricity consumption and emissions flows that remain invisible under conventional territorial accounting.

Building on this literature, this paper develops and evaluates a consumption-based approach for allocating cloud emissions. Our methodological contribution lies in adapting established consumption-based principles from trade and manufacturing domains^7^^,^^8^^,^^9^ to the distinct characteristics of cloud infrastructure, using trade flows as a proxy for physical data flows to address severe data constraints. Rather than proposing a replacement of the location-based method, this work demonstrates how consumption-based metrics can complement territorial accounting by revealing otherwise hidden cross-border flows.

Our analysis focuses on the European Union’s three largest DC hubs: Germany, Ireland, and the Netherlands, which collectively host a significant share of Europe’s cloud infrastructure. These countries represent distinct configurations of digital economies and electricity systems. Germany’s DC market is large and primarily domestic, Ireland’s is dominated by multinational cloud providers serving global clients, and the Netherlands functions as a regional interconnection hub. These differences make the trio well-suited as comparative case studies for analyzing the distribution of digital emissions and testing the feasibility of our allocation framework.

The study period, 2017–2022, captures a time period of rapid DC expansion and improving data availability in what remains a data-scarce research domain. ICT services already account for a growing fraction of electricity demand in these jurisdictions: DCs are projected to reach 28% of Ireland’s total demand by 2030^22^ and around 8% of the Netherlands’ demand.^23^ Similar trends are emerging globally. In the United States, DC electricity consumption is projected to rise from roughly 3%–5% in 2023 to 7%–12% by 2028, driven in part by the rapid deployment of artificial intelligence workloads.^24^ These developments underscore the urgency of refining how emissions from digital services are attributed and reported.

This study contributes to two intertwined research agendas. First, it extends consumption-based accounting to a new domain (cloud computing) where transboundary energy use has not yet been systematically quantified. Second, it provides comparative evidence across European DC hubs, highlighting how differences in electricity grid carbon intensity, DC efficiency, and consumption patterns shape each country’s cloud emissions profile. By identifying these asymmetries, the analysis provides a foundation for future, more equitable, mitigation strategies that recognize both hosting and consuming responsibilities in a globalized digital economy.

The consumption approach complements the emissions analysis by introducing two allocation categories alongside the domestic case (i.e., provider and user in the same country)^25^: the “export cloud,” where a domestic provider assists overseas cloud consumers (e.g., a German provider serving a user in the Netherlands), and the “import cloud,” where domestic cloud consumers utilize services hosted abroad. We apply this framework to the three major European DC hubs to answer the following questions.

• *How much does cloud computing contribute to national DC and TN’s electricity consumption and corresponding emissions when applying the consumption-based approach?*
• *What is the contribution of cloud data flows (imports and exports) to countries’ total DC and TN electricity consumption emissions using the consumption-based approach*?

The research focuses on allocating emissions from electricity consumption during the use phase in DCs and TNs (estimated using national power grid emission factors). Emissions related to direct energy use, other than electricity, are not included as they represent a limited share of DC emissions^16^ and stem primarily from on-site ancillary infrastructure and processes, e.g., emergency generators or fuel for liquid cooling, designed for limited operation.^26^ Other supply chain emissions, e.g., indirect emissions on the upstream and downstream of the supply chain, may constitute a substantial share of total digital service emissions,^16^^,^^26^ but are excluded due to limited data.^27^

The remainder of this article is organized as follows. The “results” section compares the magnitude and composition of cloud computing emissions across the three countries, disaggregating the contributions from DCs and telecommunication networks. The “discussion” evaluates the relevance of consumption-based metrics for national climate strategies, energy system planning, and the governance of digital infrastructure. The “limitations of the study” section outlines directions for future research.

## Results

**Figure 1:**
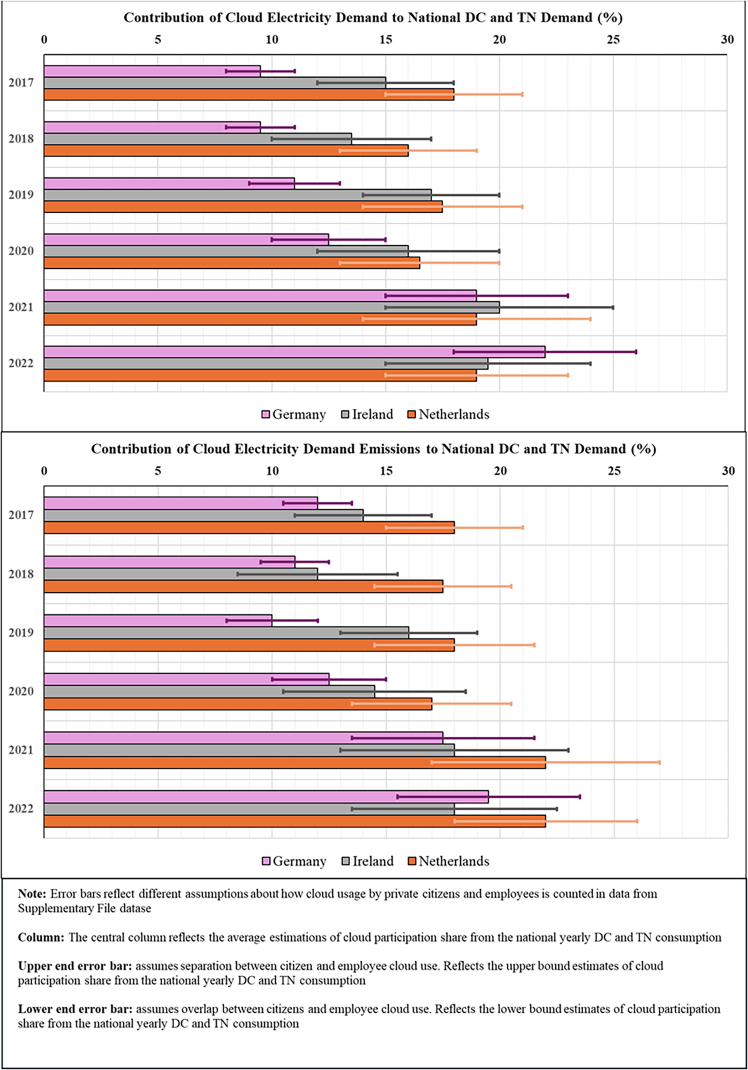
Contribution of cloud computing electricity demand and related emissions to national DC and TN electricity demand and emissions (2017–2022)

**Figure 2:**
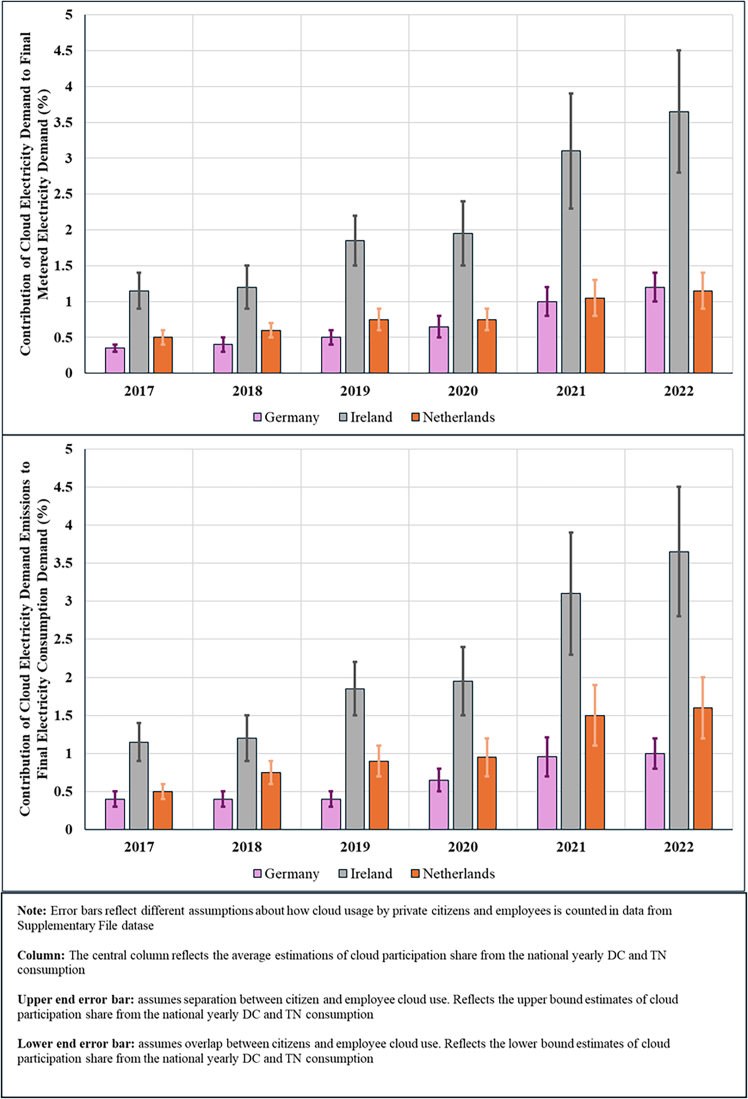
Contribution of cloud computing electricity demand and related emissions to the total national electricity demand (2017–2022)

**Figure 3:**
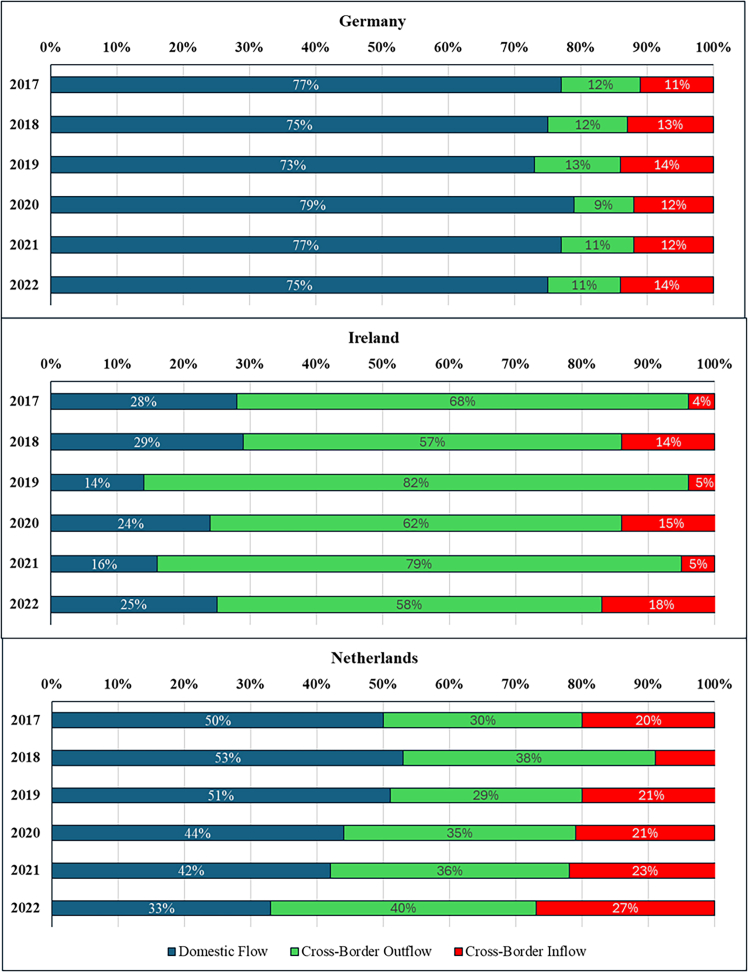
Yearly estimation of cloud computing data volume share per data flow category (2017–2022)

**Figure 4:**
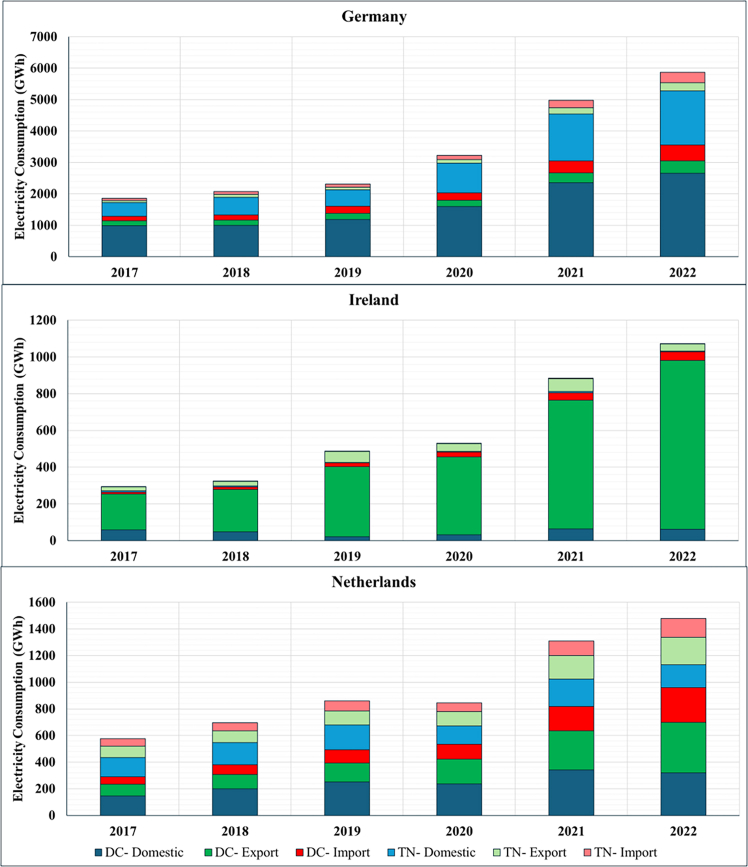
Middle range of cloud electricity consumption sub-divided by consumption type (2017–2022)

**Figure 5:**
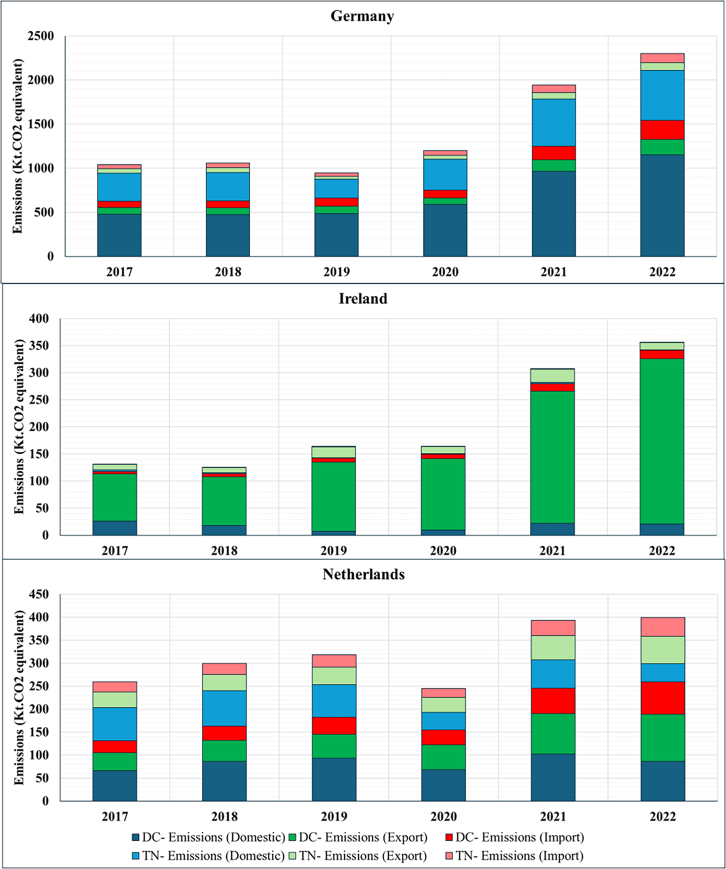
Middle range of cloud electricity consumption emissions sub-divided by consumption type (2017–2022)

**Figure 6:**
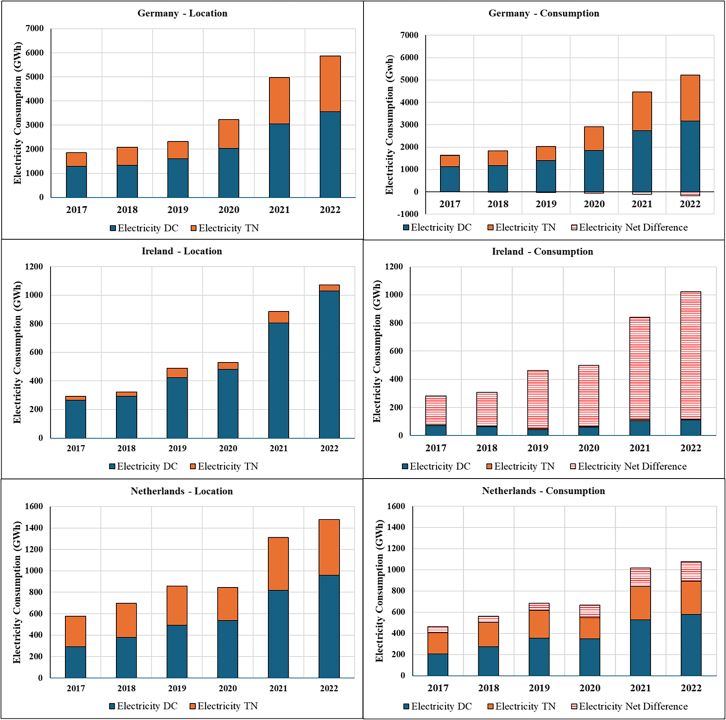
Middle range composition of electricity consumption of cloud computing services based on location and consumption-based allocation (2017–2022)

**Figure 7:**
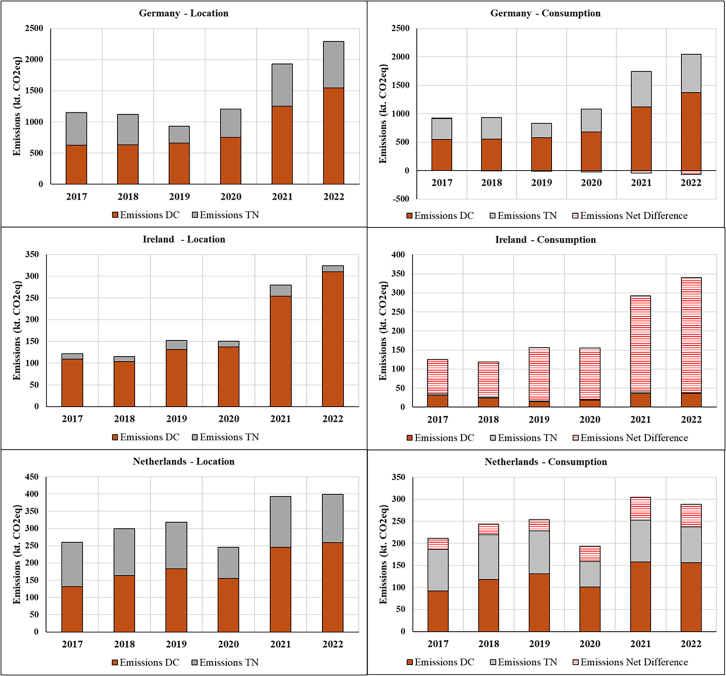
Middle range composition of electricity consumption emissions of cloud computing services based on location and consumption-based allocation (2017–2022)

The resulting estimates are presented below using consumption-based accounting, which attributes emissions to the countries where cloud services are consumed rather than where DCs are physically located. This approach differs from location-based accounting (the current standard in emission reporting) by reallocating emissions based on service demand rather than infrastructure location. Before examining the allocation differences between consumption and location-based emissions estimates, we first quantify the cloud’s total electricity demand and corresponding carbon emissions occurring within the DC and TN of the top three EU DC hubs (Germany, Ireland, and the Netherlands), along with the total electricity demand and corresponding emissions in these countries (Figures 1 and 2). We then classify electricity demand and emissions by whether these impacts are associated with domestic or cross-border data flows and reallocate impacts to countries based on location- and consumption-based accounting methods (Figures 3, 4, 5, 6, and 7).

### Contribution of cloud computing to nationwide electricity consumption and corresponding greenhouse gas emissions

Figure 1 addresses the first research question, “*How much does cloud computing contribute to national DC and TN’s electricity consumption and corresponding emissions?*” and shows that the contribution of cloud usage to the total electricity consumption and corresponding emissions of DC and TNs grew significantly between 2017 and 2022, reaching 19%–22% in 2022. This growth reflects the increased use of cloud services in the countries studied here and has been observed in both individual and enterprise cloud use, with impacts of individual cloud usage about five times higher than enterprise cloud usage impacts across all three countries and years.

Using energy (kWh/TB) and carbon intensity (kg CO_2_eq/TB) as metrics, these variations stem from fluctuations in the share of cloud data relative to the total data volume circulated in countries each year. The data volume generated by individual consumers’ use of personal cloud services increased its share of total data volume over the period, reaching ∼20% in 2022.

Figure 2 presents the estimated share of cloud in countries’ total electricity demand and carbon emissions. Germany reported an approximately 250% growth in the cloud’s contribution to national electricity consumption over the 2017–2022 period, with the cloud reaching just over 1% of total electricity demand and corresponding emissions in 2022. The Netherlands experienced a similar trend, with cloud emissions contributing approximately 1%–2% to the country’s total electricity consumption emissions in 2022. Ireland experienced the highest growth rate and participation increase, with 300% growth over the 6-year period, and cloud electricity consumption and emissions accounted for between 12% and 3.5% of total electricity demand and corresponding emissions in 2022.

### Cloud computing cross-border electricity consumption and greenhouse gas emissions

Next, we break down each country’s total cloud footprint into its data flow patterns (Figure 3), distinguishing three types of data flows: (a) domestic (origin and destination in the same country); (b) outflow or export (domestic origin and foreign destination); and (c) inflow or import (foreign origin and domestic destination). Figure 3 illustrates each country’s average composition of cloud service flows. Figures 4 and 5 showcase the breakdown by consumption type for electricity consumption and related emissions, respectively. Addressing the second question, “*What is the contribution of cloud data flows (imports and exports) to countries’ total DC and TN electricity consumption emissions using the consumption-based approach*?” the figures show that the contributions of different data flow types vary across the three countries studied here, with distinct cross-border profiles.

Germany is primarily a domestic cloud consumer, with domestic data flows contributing three times more on average (∼75%) than cross-border flows (export and import) (25%) throughout the entire period. Yet, among its cross-border cloud consumption, despite being the most significant DC hub in terms of total installed capacity in the EU,^28^ Germany experienced a gradual shift away from its initial position of a minor export surplus in 2017 to an increasing import net balance from 2018 (Figures 4 and 5). This suggests that domestic demand for cloud services outpaced domestic DC capacity expansion, increasing dependence on foreign infrastructure to meet computational needs.

Conversely, Ireland has expanded its position as a carbon exporter (∼85%). Ireland’s cloud services are cross-border-oriented, with domestic consumption accounting for only a minor share of total foreign data flows (Figures 4 and 5). This export-dominated profile reflects Ireland’s strategic positioning as a European hub for hyperscale DCs operated by firms such as Google, Microsoft, and Amazon Web Services, which primarily serve continental users rather than domestic demand. For example, Google’s large-scale facilities in Dublin are integrated into pan-European cloud infrastructure, thereby increasing cross-border data flows relative to domestically oriented traffic.

Despite handling the lowest aggregate data volume, Ireland exhibits the highest energy and carbon intensity of cloud computing (Document S2). This reflects structural constraints in the domestic power system: Ireland’s renewable share remains below the EU average, and rapid DC growth has contributed to severe supply deficits requiring increased reliance on fossil generation and high-carbon power imports.^22^

Lastly, the Netherlands exhibits a more balanced data flow profile compared to Germany and Ireland, although the export share gradually expanded during 2017–2022 (Figures 4 and 5). The Netherlands has the lowest energy and carbon intensity of cloud computing among the three countries (Document S2). Although the Dutch case comprises the most distributed data flow between the three countries, from 2021 onwards the country started to increase its net export, indicating that the hosted physical ICT infrastructure growth outpaced the domestic digital demand. Additionally, the Netherlands also has the lowest emission growth rate, with its 2022 level 240% above the 2017 baseline.

### Energy and emission development

Across all cases, gains in energy efficiency and emission factors have reduced the energy intensity of data volume (kWh/TB) for DCs and TNs on each country’s electricity grid, resulting in smaller national increases in emissions relative to electricity consumption (Document S2). Yet, the pace of improvement varies. Germany gradually flattened its electricity consumption growth relative to data volume growth: a 60% decrease in electricity intensity in 2022 compared to 2017 levels for DCs, and a 40% decrease for TNs. Ireland had the lowest reduction in DC intensity, with only a 10% decrease from 2017 levels until 2022 for DCs and a 65% decrease for TNs. The Netherlands recorded the fastest decrease in electricity consumption, with a 70% reduction in electricity intensity in 2022 relative to 2017 levels for DCs and 90% for TNs (Document S2).

Higher efficiency and increased penetration of renewables also affected the emission intensity of data volume (kg CO_2_eq/TB) for DC and TNs. Germany saw gradual efficiency gains and decoupling of emissions from electricity consumption, with a 65% decrease in DCS’s energy-use intensity in 2022 relative to 2017, and an 80% decrease for TNs. Ireland reported the lowest DC efficiency growth: a 35% decrease in carbon intensity in 2022 from 2017 levels, and a 75% decrease for TNs. In line with our results on electricity intensity, the Netherlands also showed the fastest decrease in carbon intensity—an 80% decrease in 2022 from 2017 levels for both DCs and TNs (Document S2).

Cumulatively, these energy efficiency improvements, coupled with higher renewable penetration, enabled a 55% decrease in Germany, a 40% decrease in Ireland, and a 30% decline in the Netherlands from the electricity consumption emissions stemming from cloud operations between 2018 and 2022, when compared to a counter-factual scenario with same data volume growth but without improving energy efficiency and decreasing electricity emissions intensity.

## Discussion

The consumption-based approach emphasizes the structural misalignment in how cloud computing emissions are currently assigned. Under location-based accounting, jurisdictions that host DCs bear the full responsibility for the emissions in their national inventories, even when a large portion of the services produced are consumed overseas. This creates potential equity issues: hosting jurisdictions benefit from strengthened digital infrastructure but also face local environmental impacts, while consuming countries benefit from the services without accounting for them in their climate inventories. The extent of this misalignment varies greatly: In Ireland, an estimated 85% of cloud-related emissions stem from cloud services consumed abroad, in 2022, whereas Germany’s emissions are caused mainly by domestic users (approximately 75% in 2022). Yet, its consumption increasingly depends on facilities located elsewhere.

Figures 6 and 7 break down the differences in emission inventories between the location-based and consumption-based approaches. The “net difference” (electricity net difference/emissions net difference) illustrates the total cross-border net balance (export-import) in electricity consumption and emissions national inventories when the consumption-based approach is applied. A positive value means the country is a net exporter, while a negative value means the country is a net importer. These discrepancies highlight how dominant carbon accounting models, designed around territorial emissions, raise concerns when production and consumption are geographically separated.

In national climate planning, traditional planning that projects ICT demand solely from domestic demand may underestimate actual infrastructure requirements if significant capacity serves foreign consumers. Germany exemplifies this problem: despite being the EU’s largest DC hub in terms of capacity,^28^ its consumption-based footprint shows a growing reliance on cloud services hosted abroad. The German case suggests that domestic DC capacity planning based solely on domestic demand may diverge from actual consumption patterns.

Conversely, countries like the Netherlands, with high renewable energy penetration and low grid carbon intensity, face different planning considerations. Their position as net exporters of lower-carbon cloud services (480–610 GWh net export) indicates that infrastructure investments may be partially serving foreign demand. Incorporating consumption-based metrics into national energy planning frameworks could yield more accurate demand forecasts, particularly for countries where cross-border cloud flows account for 20%–30% of total cloud activity. However, operationalizing this information poses significant challenges: consumption patterns shift dynamically with market conditions, and cloud operators do not publicly disclose the destinations of facility-level consumption.

For national policymaking, consumption-based accounting reveals potential alignment imbalances between those who benefit from infrastructure and who finance it. DC development also imposes hidden costs on hosting countries (e.g., grid reinforcement, emissions, cooling water infrastructure, and land use pressures) that are currently recovered through general planning obligations, property taxes, or electricity grid charges.^29^^,^^30^

Addressing this misalignment could involve several policy approaches, though the most practical approach is to deploy DC planning obligations at the host country’s level. Facility-level contribution rules for licensing new DCs in host jurisdictions could tie infrastructure funding obligations to expected energy consumption rather than floor area or property value. Some jurisdictions already employ peak capacity thresholds for large energy users.^31^^,^^32^ These peak capacities could be extended to large-scale DC facilities, setting enhanced developer obligations above certain capacity thresholds (e.g., >50 MW peak load), which could include.

• Scheduled contributions to grid capacity funds proportional to exceeding levels above the peak capacity
• Requirements to co-invest in renewable generation or transmission upgrades in the local grid
• Annual reporting of energy consumption DCs to inform grid planning^33^
• Conditional license renewal based on infrastructure impact assessments^31^^,^^32^

The focus on host-based policies stems from the challenges in implementing consumption-destination-based obligations. Policies targeted at the consumer demand side face three barriers: (1) DC operators and cloud providers do not disclose customer geography or workload destinations for competitive reasons; (2) consumption patterns can shift dramatically post-licensing as operators scale or repurpose DC facilities; (3) cloud workloads are dynamically distributed across facilities, making attribution or emission streams to specific DCs technically infeasible with current reporting standards.^34^ These implementation limitations suggest that any policy intervention to compensate for local impacts of cloud emissions must reference the hosting facility’s characteristics rather than modulating consumption patterns at the destination.

Nonetheless, these implementation barriers do not diminish policy value of consumption-based accounting for strategic infrastructure planning. While direct consumption-based obligations policies for DC facilities remain unlikely at the national level, transboundary cloud consumption data can inform more strategic policymaking at the international scale. Host countries can use the consumption approach to better assess the trade-offs between the local infrastructure costs and economic benefit spillovers when evaluating DC investments. The consumption-based approach opens a new scope of bilateral or multilateral policies from which host countries can coordinate with consumer countries to optimize DC allocation in order to reduce to the aggregate emissions from cloud services. To understand the potential impact of strategic cloud infrastructure imports on total emissions, Table 1 presents estimated changes in total cloud emissions when the amount of cloud imported to Germany is hosted on other grids.

**Table 1:** Regional sensitivity analysis of Germany’s 2022 cloud emissions by origin of imported cloud electricity

	Upper Range				Lower Range			
	% Range from Baseline Emission Value		Total Cloud Emissions per Scenario According to Baseline Value (kt. CO_2_eq)		% Range from Baseline Emission Value		Total Cloud Emissions per Scenario According to Baseline Value (kt. CO_2_eq)	
	Plausible Scenario	Scenario Limit	Plausible Scenario	Scenario Limit	Plausible Scenario	Scenario Limit	Plausible Scenario	Scenario Limit
(A) EU, Iceland, Norway, UK	−4%	−2%	2630	2660	−4%	−4%	1800	1800
(B) Balkans and Türkiye	+12%	+8%	3050	2950	+12%	+12%	2080	2080
(C) Eastern Europe and South Caucasus	0	−2%	2730	2660	0	0	1860	1860
(D) Middle East and North Africa	+8%	+7%	2950	2930	+8%	+8%	2020	2020
(E) Central Asia	+11%	+8%	3040	2950	+11%	+9%	2080	2030

The consumption-based approach allows for a more granular understanding of not only the reallocation of emission responsibility but also the identification of decarbonization opportunities through the strategic geographic placement of cloud services (“geographic arbitrage”). The table below showcases the case for Germany in 2022. Hosting the imported cloud amount in Germany instead would, on average and across most grids, reduce total emissions if originally hosted in Europe (∼4% lower). The hosting impact on Eastern European or Caucasian grids would, on average, be marginal and close to 0 (Table 1). Yet the impact on emission levels becomes significant if the same cloud service is hosted in grids on the Balkans, Central Asia, or the Middle East (∼8%–12% higher). In terms of policy implications, applying the consumption-based approach opens new opportunities to identify aggregate decarbonization opportunities via strategic imports of services hosted in grids with low-carbon energy availability. Table 1. Regional sensitivity analysis of Germany’s 2022 cloud emissions by origin of imported cloud electricity. This paper examines a consumption-based approach as a complementary method for allocating cross-border cloud emission flows and outlines potential policy directions for addressing geographic asymmetries in cloud demand and supply. While location-based accounting estimates the aggregate emissions from sources within a country’s territorial boundaries, the consumption-based approach reveals the geographical distribution of the demand driving those emissions. Neither method alone captures the complete picture: location-based accounting reveals where energy demand materializes and where infrastructure must respond; consumption-based accounting shows where benefits accrue and where demand policies might target.

Location-based methods underpin territorial climate commitments and international reporting obligations. Complementarily, consumption-based methods provide analytical insights into transboundary flows that may inform bilateral cooperation, corporate disclosure, and national planning. While this paper focuses on diagnosing cross-border asymmetries that location-based accounting does not capture, an important next step is integrating both approaches to inform practical decision-making. Future work could advance this discussion by developing a responsibility-sharing implementation framework to guide the allocation of climate responsibility between hosting and consuming jurisdictions.^9^

In the period examined here, cloud computing still had a relatively modest impact compared to the overall ICT sector, accounting for approximately 25% of the total data flow in 2022 (Document S2). However, the rapidly rising contribution of cloud computing to total ICT sector data flows in the three countries studied here, combined with the growing share of ICT services in these and other countries’ overall carbon footprint, creates an urgent need for policymakers to consider cloud expansion and DC performance goals alongside climate targets. Adopting a consumption-based emissions reporting criterion, in addition to current location-based accounting approaches, increases transparency and can support more strategic carbon mitigation efforts.

Considering climate targets and future power constraints, consumption-based criteria provide evidence of cross-border emission flows that may inform national climate strategies and international cooperation. Such data can support infrastructure planning and bilateral cooperation initiatives. For example, countries with carbon-intensive electricity grids and high ICT consumption might explore bilateral agreements with countries with high renewable energy shares to jointly finance low-carbon DC capacity. The importance of new allocation methods and internationally oriented approaches to digital services governance is further underscored by evolving business practices and the increasing use of tariffs to regulate international business relations in digital services.^35^

Importantly, the consumption-based approach developed and applied in this study to the case of three major DC hubs in Europe, is intended to complement, not replace, standard location-based methods, nor to prescribe normatively how responsibility should be shared between hosting and consuming jurisdictions. Developing equitable allocation frameworks that integrate both perspectives remains an important direction for future research and international policy development.^9^^,^^36^ Our methodological contribution provides a modeling foundation for such frameworks by making visible the cross-border flows that territorial accounting alone cannot capture. The two approaches address different policy questions and are most valuable when used together to provide a comprehensive understanding of emissions sources, responsibilities, and potential mitigation pathways, without presuming that either method alone adequately captures the complex responsibilities in globally interconnected digital infrastructure.^36^

While this analysis focuses on use-phase electricity consumption and related emissions from DCs and TNs, the consumption-based allocation approach developed here could be extended to other life cycle stages and environmental impact categories in future research. This would provide a more comprehensive understanding of the full environmental footprint of cross-border cloud services. Moreover, the consumption-based approach also expands the scope of future policy options for coordinating supply and demand of digital services across countries, using bilateral or multilateral agreements to strategically site DCs in ways that reduce total cloud-related emissions.

### Limitations of the study

The analysis presented here has two main limitations that could be addressed in future work. First, due to the limited availability of facility and supplier data, this work primarily utilized national averages for emission factors and DC electricity consumption. Future work should incorporate energy supplier-specific emission factors and facility-level energy demand data from DCs when they become publicly available. Likewise, industry reports, flow measurements from internet service providers (ISP), and data localization reports could improve the precision of estimates.

Second, the attribution of emissions and electricity demand to domestic and cross-border flows is based on an indirect estimate of data flows derived from trade data (i.e., domestic value generation and cross-border trade of ICT services, see Document S2 for details). In the paragraphs that follow, we discuss the implications of this and other limitations for the robustness of our results.

Limitation 1: the Dutch temporal accuracy of DC electricity consumption in 2022. In the Netherlands’ case, final figures for total DC electricity consumption for 2022 were not available at the time the analysis was completed. Based on historical Dutch trends, this study assumed a fixed growth rate of 16.4% for nationwide DC electricity consumption between 2021 and 2022. Given the COVID pandemic (2019–2021), deviations in cloud use intensity (MB/kWh) may have led to greater variations in electricity consumption than those estimated here, and consequently to larger differences in total cloud emission levels than reported. This uncertainty primarily affects the temporal precision of the results for the Netherlands in 2022.
Limitation 2: use of trade data proxy for data flows. The use of economic indicators resulted from the lack of reliable sources of data flow information (origin/destination of data volumes), which adds uncertainty to cloud emissions estimates. This reliance on economic indicators introduces potential biases, as the relationship between a cloud service’s economic value and its energy intensity is non-linear. For example, financial services, such as financial transactions, generate orders of magnitude less data traffic per euro of economic value (MB/€) than low-value, high-volume services such as video streaming or social media. Since the method assumes proportionality between cross-border flows and trade value, it overestimates the energy-per-euro ratios (MB/kWh per €) of countries specializing in high-financial-value, low-data-volume services relative to the actual data traffic their infrastructure handles. Contrastingly, our method underestimates energy-per-euro ratios in countries dominated by low-value, high-volume services.

This bias has direct implications for the interpretation of the results. Ireland, dominated by multinational cloud providers serving global enterprise and financial clients,^22^ potentially exports a higher proportion of high-value, low-volume services than the economic proxy can capture. Consequently, the study’s estimates may overstate Ireland’s export-related energy intensity: actual data on real traffic and energy consumption per euro of exported services may be lower than the estimates suggest. Conversely, Germany and the Netherlands, where cloud consumption is dominated by higher-volume, lower-value services (e.g., streaming and social media), may exhibit higher data volumes per euro than Ireland. This heterogeneity means the economic proxy may underestimate the energy intensity of their domestic and imported cloud services relative to their actual infrastructure demands.

Overall, however, the differences in countries’ data flow profiles remain significant even when accounting for the varying energy intensities of ICT services. The consistency of Ireland’s export-dominated pattern across all years examined (2017–2022) and across both upper- and lower-boundary estimates provides additional confidence in the directional finding, even if the precise quantitative estimates remain uncertain. Second, the sensitivity analyses indicate that alternative assumptions about data volume distributions in the Irish case would need to differ more substantially to reverse the relative rankings of these countries’ export orientations. While the economic proxy affects absolute values, the qualitative distinction between Ireland as a net exporter, Germany as primarily domestic, and the Netherlands as balanced remains supported by the available evidence.

Limitation 3: division between enterprise and personal cloud use. Beyond the uncertainties in the relationship between data volume and financial value, the consumption-based estimates are sensitive to additional methodological assumptions. Those assumptions primarily cluster around the total cloud volume and the division between enterprise and personal clouds. These assumptions produce divergent sensitivity patterns across countries, reflecting their underlying differences in data consumption characteristics. Germany and the Netherlands show a variation of around 35% in emission levels relative to the baseline value discussed in this paper. These differences stem from assumptions about the shares of enterprise and citizen cloud data volumes, making the estimates sensitive to the split between enterprise and household cloud data volumes. Ireland exhibits greater variation despite being modeled under the same assumptions. The country’s low domestic data-use intensity relative to its large export flows underlies this variation.

Specifically, the assumption that 40% of IT workloads are off-site (cloud-based) has been applied to estimate citizen cloud data volumes. This transposition may introduce biases when enterprise workloads generate data volumes that differ fundamentally from citizen (consumer) cloud workloads; e.g., consumer workloads may reach 100% cloud for certain activities (online gaming, video streaming), whereas traditional enterprise activities (databases, ERP systems) may reach only 40% cloud. Applying this enterprise-derived factor to model citizen behavior may introduce conservative bias in our estimates, potentially underestimating the citizen cloud footprint and volume. This choice to use the 40% off-site workload reference for citizens stemmed from the lack of sufficient studies and statistics on the composition of internet traffic for cloud consumers at the macro (sectoral) scale, leading to the use of enterprise metrics to also reflect citizen behavior.

Moreover, the sensitivity to emission factor assumptions adds another dimension of uncertainty that interacts with each country’s cross-border flow patterns. For the modeling, this paper used a counterfactual assumption about cross-border flows, in which the footprint of cross-border cloud data volume is subject to the same emission factors as domestic flows. This assumption is due to insufficient data to determine the destination country of data flows. The Document S2 provides a more detailed breakdown of how the origin regions of cloud services impact emissions estimates. This sensitivity becomes more pronounced as the share of cloud imports increases; DC hubs with lower import shares are more insensitive to fluctuations in origin. German imports originating from carbon-intensive regions such as the Balkans and Turkey would increase total cloud emissions by circa 12%; Central Asian origins would produce a similar increase of 11%, while hosting the cloud in another EU country could, on average, decrease total cloud service emissions by 4%. Conversely, Ireland’s minimal import dependence reduces the variability in origin scenarios, with most regions falling within 3% of the Irish baseline.

Future studies could overcome the limitation of economic proxies by incorporating more accurate software and traffic-based measurements. On the software side, this entails using the energy and carbon intensities of energy-intensive computational tasks (e.g., machine learning) and the overall task count, rather than data volume. Conversely, traffic flow data would require greater transparency into geographic customer distribution. Yet, this would entail overcoming practical barriers in data flows that require policy intervention. First, cloud operators do not currently disclose customer geography or workload destinations due to competitive sensitivities and concerns about revealing business relationships and market positions.^34^^,^^37^ Second, consumption patterns shift dynamically post-licensing as operators scale facilities, repurpose capacity, or redirect workloads across global infrastructure networks, making static geographic attribution technically challenging to report.^38^ Overcoming the limited data on the distribution of customer geography would likely require coordinated policy interventions across multiple levels. Some of these policies would be required at the national level, such as creating new consumption-based reporting schemes for large-scale (hyperscale) DC operators. Complementarily, some of this transparency would also rely on international cooperation to disclose customer geographies, either through bilateral data-sharing agreements between countries with significant cross-border data flows or by modeling existing multilateral instruments, such as the international energy agency’s digital energy efficiency platform.^39^

Lastly, this work focuses on historical data and should be used to evaluate past trends, not to develop projections. Based on the top-down approach employed here, past (i.e., 2017–2022) DC and telecommunication network characteristics are reflected in energy use and emissions data with reasonable accuracy; however, future changes may not be well captured in simple linear or exponential extrapolations. Bottom-up estimates of DC energy use could better account for the energy use and emissions impacts of rapidly evolving approaches to DC operations optimization, cooling, and heat-reuse technologies, increasingly efficient servers and processors, as well as the growing use of accelerated servers for artificial intelligence services. While bottom-up approaches have their own challenges, including reliance on substantial knowledge of the installed DC equipment base, they can be more easily adjusted through periodic data updates to reflect technology changes and improved component-level data availability.

## Resource availability

### Lead contact

Further information and requests for resources should be directed to and will be fulfilled by the lead contact, Ian Varela Soares (ivsaa@connect.edu.hk or ian.varela93@outlook.com).

### Materials availability

This study did not generate new unique materials.

### Data and code availability

• Datasets: The dataset supporting this study is available in the key resources table and the Document S2 (paper doi). No data were deposited in a public repository; the full input dataset and results are provided as a supplementary Excel file accompanying this paper.
• Code: All calculations were performed in Microsoft Excel. No custom code was generated. The annotated Excel workbook with full calculation steps is provided in the Document S2 (paper doi) and is available from the lead contact upon reasonable request.
• Any additional information required to reanalyze the data reported in this paper is available from the lead contact upon request.

## Acknowledgments

I.V.S. gratefully acknowledges support from the Hong Kong Research Grants Council through the Hong Kong PhD Fellowship Scheme.

## Author contributions

Conceptualization, I.V.S., M.Y., and M.M.K.; data curation, all authors; formal analysis, I.V.S.; investigation, I.V.S., M.Y., and M.M.K.; methodology and supervision, all authors.; validation, all authors; visualization, I.V.S.; writing – original draft, I.V.S.; writing – review and editing, all authors.

## Declaration of interests

The authors declare no competing interests.

## STAR★Methods

### Key resources table

**Table:** 

REAGENT or RESOURCE	SOURCE	IDENTIFIER
**Deposited data**		

Dataset with reference values	This paper	paper doi

**Software and algorithms**		

Microsoft Excel (365 version)	Microsoft Corporation	www.microsoft.com

**Other**		

Document S2 outlining the calculation in full detail	This paper	paper doi

### Experimental model and study participant details

Omitted as our study does not involve biological models.

### Method details

#### Analytical framework

Since the work focuses on emissions allocated to national emission inventories, the ICT sector is examined from a service-supply perspective. The ICT sector is segmented into three domains^10^^,^^11^:

1. Telecommunication networks: Data transmission and network traffic (e.g., broadband and mobile networks).
2. Data centers: On-site and off-site IT workloads, facilities, and the computer servers running within.
3. End-user devices: Encompassing consumers' electronic devices.

The research only encompasses the first two components of the digital infrastructure, e.g., the networks and data centers. End-user devices are outside the scope of this analysis since the focus is on use-phase electricity-related emissions from cloud computing services. Figure S1 outlines the scope of the analysis.

This distinction between operational (consumption) and supply chain emissions is relevant because our analysis focuses on emissions related to the use phase electricity consumption of the whole (service supply) of DC and TNs. In an organization-centered analysis, DC electricity consumption emissions could be upstream emissions from a cloud provider, making it difficult to isolate the industry's emissions without double-counting or distorting operational-to-supply-chain emission ratios.^40^^,^^41^ To compute emissions from electricity consumption, we use historical power grid emission factors from official government sources that represent average grid electricity consumption between 2017 and 2022.

For estimating DC and TN electricity consumption emissions, we use the GHG Protocol definition of the location-based method. It should be noted that the ‘location-based’ terminology from the GHG Protocol refers to the method for calculating Scope 2 emissions, which is distinct from the ‘location-based’ allocation principle – the former refers to a calculation process, the latter to an emission assignment principle. The GHG Protocol advises the location-based method as more appropriate for measuring the Scope 2 emissions of energy-intensive sectors. Second, data availability limits the feasibility of market-based calculations. For example, while the percentage of renewables supplied to data centers is known for the Netherlands,^42^ Germany, and Ireland, only partial yearly-average data at national on renewables supply to data centers are available.^43^ The table below illustrates this difference, distinguishing between the emissions responsibility of the cloud provider (the DC operator) and the cloud user (the service consumer) between the location and consumption-based approaches:

**Table:** 

Location of cloud computing service…:		Responsible for electricity consumption emissions in line with:	
…provider	…user	Location-based accounting	Consumption-based allocation
Germany	Germany	Germany	Germany
Germany	Netherlands	Germany	Netherlands

This distinction allows the cloud footprint to be categorized into three categories. The first is domestic emissions (domestic cloud) when the cloud providers and users are in the same country. The second is added emissions (export cloud), which occur when domestic cloud providers export services to foreign users. Third is imported emissions, which occur when domestic cloud users import cloud services from foreign providers.

#### Calculations

For the calculations, readers are encouraged to refer to the Document S2s for a more detailed assessment of the methodology and the plausibility assessment of the estimations (Document S1), a complementary breakdown of the DC and TN participation in the total electricity consumption, as well as cloud data volumes (Document S2), the reference graphs and tables for the estimation ranges (max/min value), Sensitivity Analysis (Document S2), and a conversion unit reference. The Document S1 provides substantial additions to calculation steps and values (energy and carbon historical intensity ratios for DC and TN, as well as cloud data volume amounts).

There are three main parts to the estimation of cloud computing's climate impacts:

1. In the first step, it is necessary to estimate energy demand and emissions from cloud computing across the DC and TN infrastructure for each country case (Germany, Ireland, and the Netherlands). This was done by isolating the share of total DC and TN energy consumption and emissions attributable to cloud computing services, specifically by aggregating cloud data used by employees and citizens at the country level.
2. Then, it is necessary to separate the share (%) of the cloud footprint that stems from cross-border cloud data flow. This estimation relies on data flow traffic (TB) and each country's international trade of ICT services.
3. Based on the cross-border data flow, energy (kWh/TB) and carbon (CO_2_eq/TB) intensities were derived to calculate each country's energy consumption and corresponding emissions of cloud services over DC and TN. Figure S2 simplifies the main parts of the calculation.

Details of each step are provided in Document S1. Before proceeding, a final significant caveat is that this study does not discuss the calculation of energy consumption for computational tasks. A growing body of literature criticizes the limitations of estimating future environmental footprints using average intensity ratios of data volume, suggesting that the footprint should be calculated from computational tasks.^17^^,^^19^ Nevertheless, this work uses historical data and provides historical estimates, not projections. As such, the energy consumption, GHG emissions, and total data volume are known in advance.

Part 1: Share of cloud electricity consumption and corresponding emissions over the whole data center and telecommunication network sectors in a specific country. This is achieved by estimating energy and carbon intensity ratios and using them to calculate cloud energy consumption and the corresponding emissions. The energy intensity ratio (kWh/TB) is calculated by dividing the average aggregate electricity consumption (kWh) by the total data volume (TB) for DCs and TNs in each country and year. A similar process entails the carbon intensity ratio (kt CO_2_eq/TB). The ratio calculations do not differentiate between DCs and TNs of varying energy intensities. The data used for this part were obtained from country-level averages of electricity consumption (kWh), emissions (kt CO_2_eq), and data flow (TB) provided by the governments. This first step initiates with the energy intensity (EI) ratio:

$$EI_{\left(DC\;|\;TN\right)}=\frac{p_{i}}{DV_{i}}$$

Where p_i_ is the electricity consumption (kWh) and DV_i_ is the total data volume (TB) for TNs or DCs for country *i*. The data can be found in Table S7 of Document S2.

This intensity ratio is then calculated for the national cloud data volume. Part 1 of the calculation is divided into two steps because it addresses two levels of granularity: country-level (energy and emissions) and citizen- and employee-level (cloud adoption). To overcome differences in granularity and estimate the proportion of total DC and TN energy consumption attributable to the cloud, it is necessary to perform a series of calculations that aggregate data from all citizen and employee levels to the national level. These energy intensity ratios are used as a baseline for calculating the carbon emissions (E_s_) (Scope 2) from energy consumption for the countries:

$$E_{s2i}=\sum_{i}p_{i}*ef_{i}*GWP$$

Where *ef*_i_ is the national emission factor of average grid electricity (kg CO_2_eq/kWh) and GWP is the global warming potential (for CO_2_ = 1).

To calculate the citizen and employee data, the work adapts the method proposed by Snelson et al.^21^ on estimating the total number of cloud users (enterprise) for each country and year. However, this work expands the method to include (a) public employees and (b) individual cloud users (citizens). The calculation is done by first estimating the total number of cloud users: employees according to their average headcount (for public and private enterprise users)^42^^,^^43^^,^^44^^,^^45^^,^^46^ and the number of citizens with internet access using cloud services (for citizens).^47^^,^^48^ It is assumed that the data intensity of the cloud differs between citizen and employee use, and that the total cloud data aggregates both citizen and employee use. To separate citizen and employee data volumes, this work uses data from Cisco, indicating that the share of employee data flow is approximately 16% of the total data volume yearly between 2017 and 2023, and 84% for citizen internet data.^49^^,^^50^^,^^51^ By multiplying the share (%) of citizen and employee data volume by the total volume, it is possible to estimate the amount (TB) of enterprise and individual data circulated.

With estimates of data volume for each use type, the next step is to calculate the data-use intensity per use type (TB/employee or citizen). The citizen and employee data volumes are then used with the total number of cloud users to estimate the general data use intensity for employees (TB/employee) and citizens (TB/citizen) in the country. Once the total number of cloud users is estimated, the total cloud data volume can be calculated. The total data volume for the ICT sector, including cloud, was obtained from national government statistics datasets, as used in Part 1, and compared with the International Telecommunication Union dataset.^52^

At this stage, employee- and citizen-level data are aggregated at the country level, enabling estimation of the total cloud data flow (TB). The last step is to isolate the share of cloud data flow from the total data flow. This work used the IT workload distribution historical estimations from the Uptime’s Institute Data Center Survey.^53^^,^^54^ The average cloud proportion of the total IT workload grew gradually from 40% in 2018 to 52% in 2023.^53^^,^^54^

For the aggregate cloud flow, Eurostat^44^ does not clarify the relationship between enterprise and individual datasets, i.e., whether the individual cloud adoption dataset includes or excludes the enterprise adoption dataset. To address potential double-counting risks, the work applies a boundary estimation range: (a) the upper boundary, when there is no double-counting of enterprise cloud as part of the individual cloud (citizen + employee); and (b) the lower boundary, when there is double-counting of enterprise cloud as part of the individual cloud (citizen - employee). The ‘results’ section for the net balance uses a mid-range (average) estimation between the boundaries, while the upper and lower ranges are presented in Table S10 of Document S2.

Part 2: Share of cloud data flow per type (domestic / export /import) over the total cloud data flow. To separate the data flow, it is necessary to break down the cloud footprint by consumption: the amount hosted in the country (domestic), the amount produced domestically for international consumers (exported), and the amount imported from overseas data centers. Since this work uses data intensity, the primary process requires breaking down the total cloud flow by data-flow category (domestic/export/import). More details can be found in Table S8 of Document S2.

To break down the cloud data flow into these categories, with cross-border data flows divided into exports and imports, this work adapts the typology of international trade in manufactured goods.^55^ Thus, the cross-border cloud data flow is estimated based on the economic value of information and communication services:

• The cloud cross-border data flow is calculated based on the proportion of the economic value (EUR) from the international trade of ICT services vis-à-vis the national account turnover of the ICT services

The first step is to estimate the share of international trade in ICT services within the total ICT sector turnover. This estimation assesses the relative economic significance of cross-border trade activities:

• Share (%) from the aggregate of total transactions in information and communication (Export + Import) over the total turnover of the ICT services (EUR)

To calculate the economic significance of cloud computing, specifically, this work assumes that:

• Cross-border ICT trade flows in terms of economic value and cloud cross-border data flows in terms of data volume are directly proportional
• The historical economic net balance of trade (exports-imports) for a given year is the same proportion as that of all ICT services for that same year.

Using the share (%) of cross-border turnover for ICT services, it is possible to calculate the amount of cross-border cloud flow relative to the total cloud flow for that specific country and year (TB). These calculations are summarized below. First, set the total cloud electricity consumption for country *i* across data volume range *j* (upper, middle, or lower range):

$$PC_{i,j}=\sum_{i}CU_{j}*\;EI_{i\left(TN/DC\right)}\text{,}$$

where *CUj* is the cloud data volume (TB) for the specified range. The cloud emissions are calculated analogously:

$$EC_{is2,j}=\sum_{i}{CU}_{j}*EI_{s2i\left(TN/DC\right)}$$

Cross-border data flows, CB_ES, are estimated based on the economic significance of international ICT services trade:

$$CB_{ES}=\frac{t_{I}}{t_{T}}\text{,}$$

where t_I_ is the international ICT services trade turnover and t_T_ is the total ICT services turnover (billion EUR)

Part 3: Domestic and cross-border energy consumption and corresponding emissions under the consumption-based allocation. The data flow amount (TB) for each country and year is then multiplied by the electricity intensity (kWh/TB) and emission intensity (kgCO_2_eq/TB) ratios calculated at the beginning of Part 1. These ratios are used in Part 3 to estimate the electricity consumption and corresponding emissions. Lastly, the electricity consumption and GHG emissions of DC and TN are combined to determine the total environmental footprint of cloud computing, considering the net balance of cross-border data flows.

This ratio is applied to the total cloud data volume to estimate domestic, export, and import components. The cloud data volume per category *e,f,g* (domestic/export/import) is:

$$DV_{CB\left(e,f,g\right)}=CB_{ES}*CU_{j}$$

Electricity consumption and emissions for each flow category are calculated by multiplying *DV*_*CB*_ by the respective energy and emission intensity ratios.

### Quantification and statistical analysis

The sensitivity analysis section in Table S11 of the Document S2 breaks down the variations in estimates across upper- and lower-bound ranges for cloud emissions. No inferential statistical tests were performed; the analysis is based on established national-level activity data. Uncertainty is characterized using upper- and lower-bound estimation ranges, reported as the spread around the mid-range value. The mid-range is the arithmetic mean of the upper and lower bounds.

All reported ranges represent the full extent of uncertainty across the methodological assumptions detailed in Document S1. The value of n corresponds to the number of country-year observations (n = 3 countries × 6 years = 18 per boundary scenario).

### Additional resources

The results were generated using Microsoft Excel. Detailed information about the calculations can be found in the supplementary PDF file (Document S1). Details about the values can be found in the supplementary Excel file (Document S2).

## Supplemental information

Document S1. Supplementary figures, method details, source documentation, and sensitivity analyses supporting the cloud-emissions study Includes Tables S1–S6.

Document S2. Supplementary values used for all the calculations throughout the paper, including main inputs and outputs Includes Tables S7–S11.

## References

[1] Liu F., Tong J., Mao J., Bohn R., Messina J., Badger L., Leaf D. (2011).

[2] Montevecchi F., Stickler T., Hintemann R., Hinterholzer S. (2020). https://digital-strategy.ec.europa.eu/en/library/energy-efficient-cloud-computing-technologies-and-policies-eco-friendly-cloud-market.

[3] Dietrich J. (2023). https://journal.uptimeinstitute.com/accounting-for-digital-infrastructure-ghg-emissions/.

[4] Bundesministerium der Justiz (2023). https://www.gesetze-im-internet.de/enefg/BJNR1350B0023.html.

[5] Dodd N., Alfieri F., De Oliveira Gama Caldas M.N., Maya-Drysdale L., Viegand J., Flucker S., Tozer R., Whitehead B., Wu A., Brocklehurst F. (2020).

[6] Walden I., Millard C. (2021). Cloud Computing Law.

[7] Peters G.P., Hertwich E.G. (2008). CO2 Embodied in International Trade with Implications for Global Climate Policy. Environ. Sci. Technol..

[8] Palm V., Wood R., Berglund M., Dawkins E., Finnveden G., Schmidt S., Steinbach N. (2019). Environmental pressures from Swedish consumption -- a hybrid multi-regional input-output approach. J. Clean. Prod..

[9] Engström E., Larsson M., Bradley K., Dotterud Leiren M., Ejelöv E., Finnveden G., Haugs Langvik K., Heinonen J., Larsson J., Thøgersen J. (2024). https://www.norden.org/en/publication/policy-options-reducing-consumption-based-emissions.

[10] Masanet E., Shehabi A., Lei N., Smith S., Koomey J. (2020). Recalibrating Global Data Center Energy-Use Estimates. Science.

[11] Malmodin J., Lundén D. (2018). The Energy and Carbon Footprint of the Global ICT and E&M Sectors 2010--2015. Sustainability.

[12] Malmodin J., Lövehagen N., Bergmark P., Lundén D. (2024). ICT sector electricity consumption and greenhouse gas emissions -- 2020 outcome. Telecomm. Policy.

[13] Mell P., Grance T. (2011). https://nvlpubs.nist.gov/nistpubs/Legacy/SP/nistspecialpublication800-145.pdf.

[14] Lundén D., Malmodin J., Bergmark P., Lövehagen N. (2022). Electricity Consumption and Operational Carbon Emissions of European Telecom Network Operators. Sustainability.

[15] Hiekkanen K., Seppäläinen T., Ylhäinen I. (2021). Energy and electricity consumption of the information economy sector in Finland. https://www.etla.fi/wp-content/uploads/ETLA-Raportit-Reports-107.pdf.

[16] van der Vorst T., Massop M., Smetink A., Kleter S. (2023). De digitale voetafdruk: Emissies van de digitale sector in Nederland in (toekomst) perspectief. https://www.rijksoverheid.nl/documenten/rapporten/2023/09/28/dialogic-de-digitale-voetafdruk-emissies-van-de-digitale-sector-in-nederland-in-toekomst-perspectief.

[17] Kamiya G., Bertoldi P. (2024). https://data.europa.eu/doi/10.2760/706491.

[18] Carbon Trust & Global e-Sustainability Initiative (2017). ICT Sector Guidance built on the GHG Protocol Product Life Cycle Accounting and Reporting Standard. https://ghgprotocol.org/guidance-built-ghg-protocol.

[19] Guennebaud G., Bugeau A. (2024). Energy consumption of data transfer: Intensity indicators versus absolute estimates. J. Ind. Ecol..

[20] Mytton D., Lundén D., Malmodin J. (2024). Network energy use not directly proportional to data volume: The power model approach for more reliable network energy consumption calculations. J. Ind. Ecol..

[21] Snelson S., Cilauro F., Bruni R., Woodhard R., Burton D. (2024). https://digital-strategy.ec.europa.eu/en/library/data-flow-and-economic-value-eu-framework-modelling-update-and-data-collection.

[22] EirGrid & System Operator Northern Ireland (2022). https://cms.eirgrid.ie/sites/default/files/publications/EirGrid_SONI_Ireland_Capacity_Outlook_2022-2031.pdf.

[23] BloombergNEF (2021). https://www.eaton.com/content/dam/eaton/company/news-insights/energy-transition/documents/bnef-eatonstatkraft-data-center-study-en-us.pdf.

[24] Shehabi A., Newkirk A., Smith S., Hubbard A., Nuoa L., Siddik M., Holecek B., Koomeu J., Masanet E., Dale S. (2024). Energy Analysis and Environmental Impacts Division.

[25] World Resources Institute (WRI) (2015). GHG Protocol Scope 2 Guidance: An Amendment to the GHG Protocol Corporate Standard. Greenhouse Gas Protocol. https://ghgprotocol.org/sites/default/files/2023-03/Scope%202%20Guidance.pdf.

[26] Ecorys (2023). Economisch Belang Digitale Infrastructuur. https://www.ecorys.com/app/uploads/2019/02/Ecorys-rapport-Economisch-Belang-Digitale-Infrastructuur.pdf.

[27] Mytton D. (2020). Hiding greenhouse gas emissions in the cloud. Nat. Clim. Change.

[28] German Data Centers (2023). DataCenter Outlook Germany. https://www.germandatacenters.com/fileadmin/documents/publications/GDA_Datacenter-Outlook-Germany_23-24.pdf.

[29] Ofgem (UK Office of Gas and Electricity Markets) (2023). https://www.ofgem.gov.uk/sites/default/files/2024-04/Major%20Connections%20Governance%20Document%20V1.2.pdf.

[30] Ling C., Yang Q., Wang Q., Bartocci P., Jiang L., Xu Z., Wang L. (2024). A comprehensive consumption-based carbon accounting framework for power systems towards low-carbon transition. Renew. Sustain. Energy Rev..

[31] Rankl F. (2024). https://commonslibrary.parliament.uk/research-briefings/cbp-7200/.

[32] European Comission (2024). Commission Delegated Regulation (EU) 2024/1364 of 14 March 2024 on the first phase of the establishment of a common Union rating scheme for data centres. OJ L, 2024/1364. https://eur-lex.europa.eu/eli/reg_del/2024/1364/oj.

[33] Buffie N.E. (2025). https://www.congress.gov/crs-product/R48583.

[34] Cory N., Dascoli L. (2021). https://www2.itif.org/2021-data-localization.pdf.

[35] Vat Calc (2025). US President Trump Digital Services Taxes reciprocal tariff review. https://www.vatcalc.com/global/us-president-trump-targets-digital-services-taxes-.

[36] Steidinger K., Lininger C., Meyer L., Muñoz P., Schinko T. (2016). Multiple carbon accounting to support just and effective climate policies. Nat. Clim. Change.

[37] Mytton D. (2021). Data centre water consumption. npj Clean Water.

[38] Acun B., Lee B., Kazhamiaka F., Maeng K., Gupta U., Chakkaravarthy M., Brooks D. (2023).

[39] International Energy Agency (2022).

[40] Gupta U., Kim Y., Lee S., Tse J., Lee H., Wei G., Brooks D., Wu C. (2021). IEEE International Symposium on High-Performance Computer Architecture (HPCA).

[41] Gupta U., Elgamal M., Hills G., Wei G., Lee H., Brooks D., Wu C. (2022). ISCA: Proceedings of the Chart Annual International Symposium on Computer Architecture.

[42] Dutch Data Center Association (DDA) (2021). State of Dutch Data Centers 2020. https://www.dutchdatacenters.nl/en/publications/state-of-the-dutch-data-centers-2020/.

[43] Sustainable Energy Authority of Ireland (SEAI) (2024). https://www.seai.ie/data-and-insights/seai-statistics/renewables#:%7E:text=In%202020%20the%20overall%20renewable,thirds%20(66.8%25)%20in%202019.%22.

[44] Eurostat (2023). Cloud computing services by size class of enterprise dataset.

[45] Central Bureau of Statistics (CBS) (2024). https://opendata.cbs.nl/#/CBS/en/dataset/81433ENG/table?ts=1715081124302.

[46] Central Statistics Office (CSO) dataset (2024). https://www.cso.ie/en/releasesandpublications/ep/p-lfs/labourforcesurveyquarter22022/employment/.

[47] Central Statistics Office (CSO) dataset (2024).

[48] German Federal Statistical Office Persons in Employment: Germany, Quarters, Domestic Concept/National Concept, Original and Adjusted Data Dataset. German Federal Government. https://www-genesis.destatis.de/genesis/online?sequenz=statistikTabellen&selectionname=13321&language=en#abreadcrumb.

[49] Eurostat (2024). Households - Level of Internet Access dataset.

[50] Eurostat (2024). Population on 1 January dataset.

[51] Cisco (2017). Cisco Visual Networking Index: Forecast and Methodology, 2016--2021. https://www.reinvention.be/webhdfs/v1/docs/complete-white-paper-c11-481360.pdf.

[52] International Telecommunication Union (ITU) (2024). Data Traffic Datasets. https://datahub.itu.int/indicators/.

[53] Uptime Institute (2023). Accounting for digital infrastructure GHG emissions. https://journal.uptimeinstitute.com/accounting-for-digital-infrastructure-ghg-emissions/.

[54] Uptime Institute (2023). Uptime Institute Global Data Center Survey 2023. https://uptimeinstitute.com/resources/research-and-reports/uptime-institute-global-data-center-survey-results-2023.

[55] Hasanbeigi A., Darwili A. (2022).

