# Supplementary material for: Estimating cross-border cloud computing emissions: A consumption-based approach applied to major European data center hubs
Source: iScience. 2026 May 22;29(6):116061. doi: 10.1016/j.isci.2026.116061 (PMC13224008; doi:10.1016/j.isci.2026.116061)
Supplement: Document S1. Supplementary figures, method details, source documentation, and sensitivity analyses supporting the cloud-emissions study — Includes Tables S1–S6. [file mmc1.pdf]

iScience, Volume *29*

Supplemental information

Estimating cross-border cloud computing emissions: A consumption-based approach applied to major European data center hubs

Ian V. Soares, Anna Furberg, Shoaib Azizi, Göran Finnveden, Masaru Yarime, and Magdalena M. Klemun

Figure S1. Analysis Delimitation

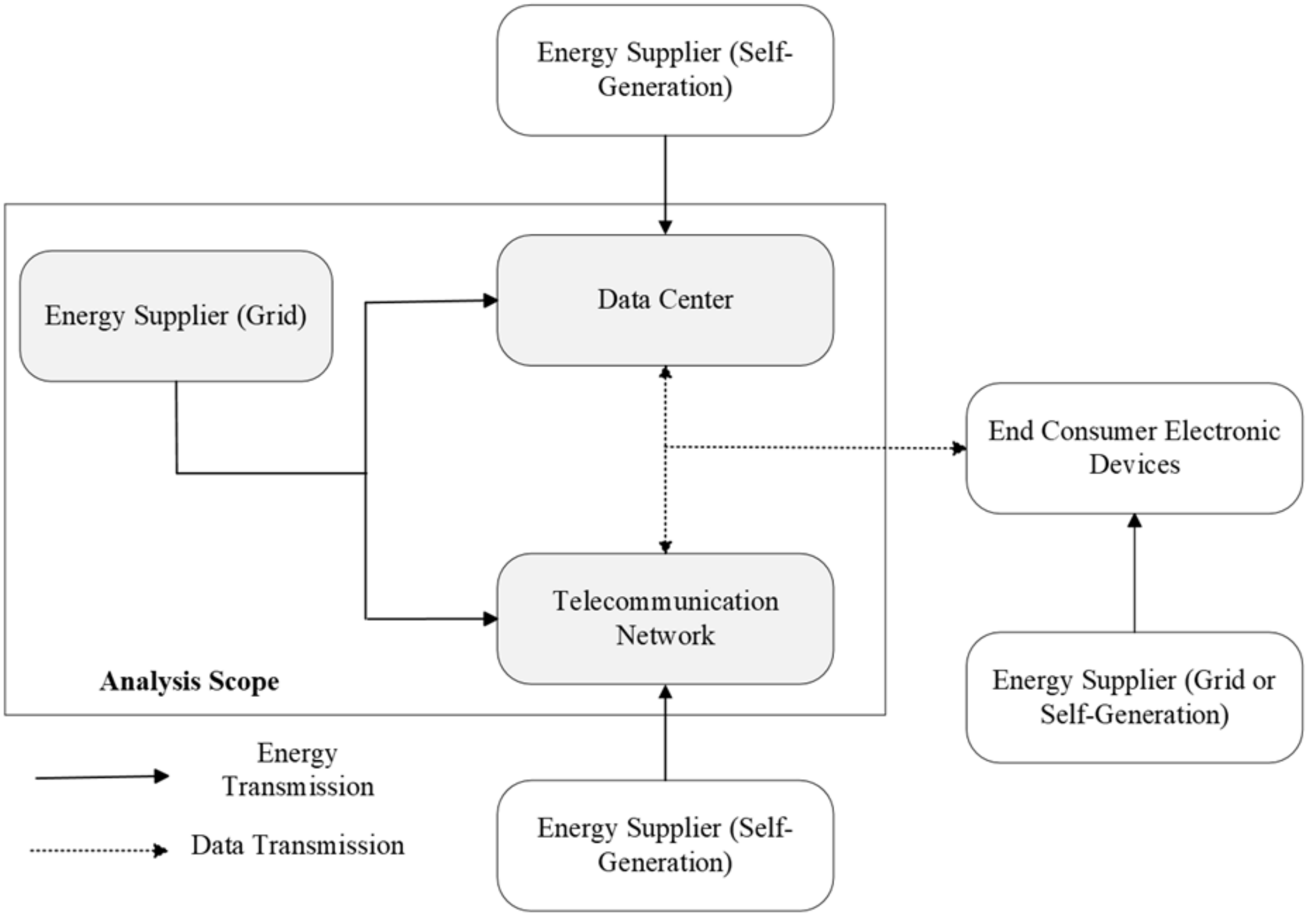

Diagram for the study boundary of cloud Scope 2 emissions for reference to the 'Method Details' section.

Figure S2. Main Calculation Parts for Estimating Consumption-based Allocation of Cloud Computing

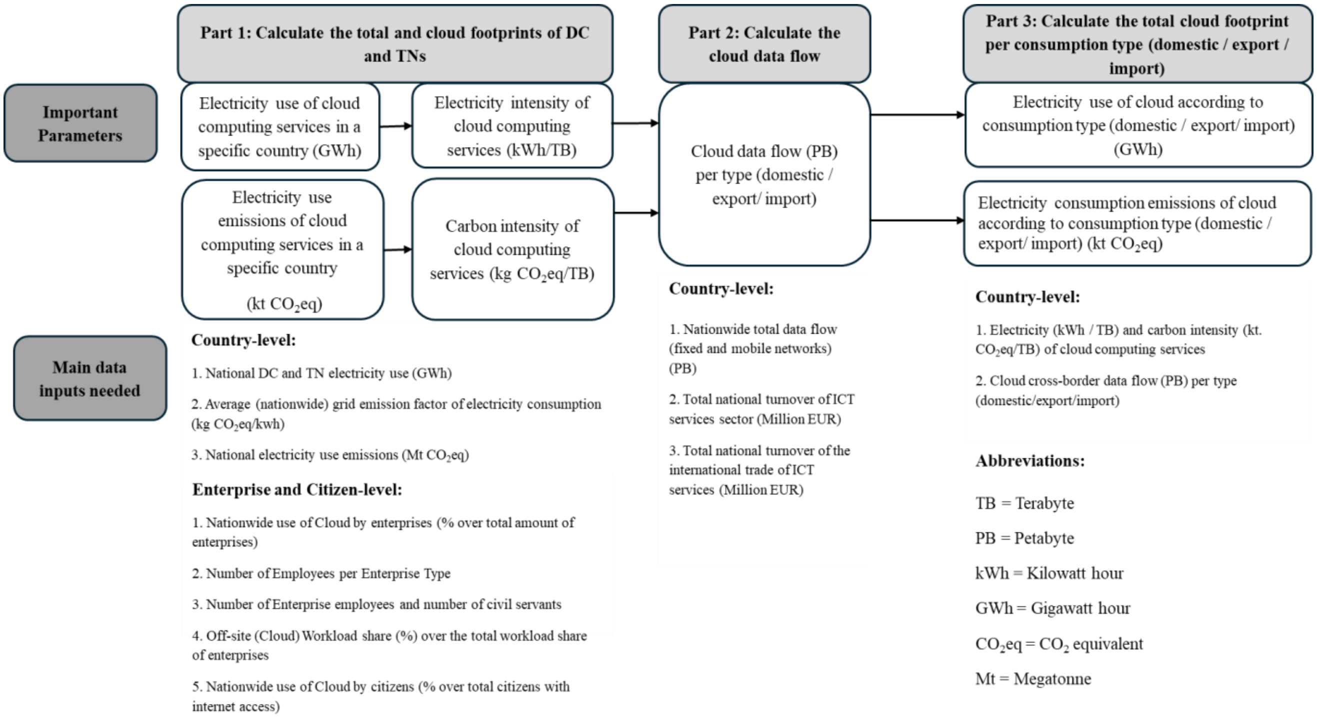

Diagram summarizing the main calculation steps for the 'Method Details' section.

Methods S1

Document S1. Supplementary figures, method details, source documentation, and sensitivity analyses supporting the cloud-emissions study.

The document presents the data sources and calculation procedures outlined in the methodology. This comprises three elements: (1) first, the data sources; (2) second, the breakdown of calculation methods; and (3) a comparison of the outputs from the calculation method proposed in this work with other existing estimations in literature –which will be labeled as the plausibility check. The main data sources used in the work are described in the table below:

Table S1. Main Data Sources and Description per Case

Data Description/Source	Country		
	Germany	Netherlands	Ireland
Energy DC (GWh) Data Description	Total Metered Electricity Consumption of Data Centers (Nationwide) (2017-2022)		
	Total Metered Electricity Consumption (Nationwide) (2017-2022)		
Energy DC Data Source	Data Center: Hintemann et al. [57]	Central Bureau of Statistics (CBS)[59]	Central Statistics Office (CSO) [60]
	Electricity: Electricity market data portal from the Federal Network Agency – SMARD [58]		
Energy TN (GWh) Data Description	Total Metered Electricity Consumption (Nationwide) of a large telecommunication operator (2017-2022)		
Energy TN Data Source	Deutsche Telekom[61—68]	KPN[69-76]	Calculations based on Virgin Media and Virgin Media O_2[77-80]
GHG Emission (Kt. CO_2eq) from Electricity Consumption Data Description	Emission Factor of Consumed Grid Electricity (Nationwide Average) (2017-2022)		
	National GHG Emissions from Grid Electricity Consumption (2017-2022) (*electricity consumption only, no heating or cooling)		
GHG Emission Data Source	Emission Factor: German Environmental Agency[81]	Emission Factor: CBS[82]	Emission Factor: Sustainable Energy Authority of Ireland - SEAI[83]
	National GHG Emissions from Electricity Consumption: German Environmental Agency[84]	National GHG Emissions from Electricity Consumption: CBS[85]	National GHG Emissions from Electricity Consumption: SEAI[86]
Data Volume (PB) Description	Yearly Total Data Volume of Fixed and Mobile Networks		
Data Volume Source	German Federal Network Agency[87-90]	Mobile Networks: Consumer and Market Authority (ACM)[95] Fixed Network: 2016 Baseline and annual growth rate calculated based on TU Eindhoven and Dialogic; and van der Vorst[96,97]	Commission for Communications Regulation[98]
	German Association of Telecommunications and Value-Added Services Providers (VATM) and Dialog Consult[91-94]		

	International Telecommunications Union (ITU) (2024)[40]		
Employment Data Description (Number of Employees)	Total Employees (2017-2022)		
	Enterprise Employees and Public Administration Employees (2017-2022)		
Employment Data Source	Total Employees – German Federal Statistics Office[37]	Central Bureau of Statistics (CBS)[34]	Central Statistics Office (CSO)[35]
	Public Administration Employees - – German Federal Statistics Office[99]		
Demographic Data Description (Number of Citizens)	Population (2017-2022)		
	Percentage of the Population with Internet Access (2017-2022)		
Demographic Data Source (Citizen)	Eurostat[38,39]		
Cloud Adoption Description (% Citizen / Employee)	Average yearly percentage of cloud adoption per total enterprises per country (2017-2022)		
	Average yearly percentage of cloud adoption individuals with internet access per country (2017-2020), and estimations of cloud adoption from internet users for 2021 and 2022 calculated based on Eurostat (* U_i and U_c calculations below)		
Cloud Adoption Source (Employee)	Eurostat[33,100]		
Cloud Adoption Source (Citizen)	Eurostat[101]		
Internet Employee Data	Percentage of enterprise data out of total internet traffic (2017-2022)		
Internet Citizen Consumer (%) Data	Percentage of consumer (citizen) data out of total internet traffic (2017-2022)		
Internet Data Source	Cisco Visual Networking Index[40] and Cisco Global Internet Report[102]		
Cloud Use (%) Description	Average yearly percentage of enterprise IT workload that happens outside of enterprise on-site data centers (2017-2022)		
Cloud Use Source	Uptime Institute Global Data Center Survey[42,43]		
Trade Data Description (Billion EUR)	International Trade (Export/Import/Net Balance) of ICT Services (2017-2022)		
Trade Data Source	Borderstep Institute and German Federal Bank[103,104]	Central Bureau of Statistics (CBS)[105,106]	Central Statistics Office (CSO)[107]

Part 1 of the calculation is divided into two steps because, as mentioned in the methodology, this part addresses two levels of granularity: country-level (energy and emissions) and citizen- and enterprise-level (cloud adoption). The latter, which required a series of calculations, was necessary because data on national-level cloud adoption were not available. Step 1 of Part 1 details the calculations for country-level data of DCs and TNs regarding their environmental footprint, whereas Step 2 of Part 1 details the cloud data volume (amount and intensities) calculations, aggregating data on citizen and enterprise-level cloud adoption to the national level, as part of the total national data volume.

To facilitate logical understanding of the calculation, this work will use 2017 data from the Netherlands as an example to evaluate the method, helping readers map out the reasoning. This calculation will compare the outputs with the estimates proposed in other works on ICT energy and emissions footprints whenever possible. Whenever the data input required for calculation was obtained from an external source, it will be indicated as the data source reference. It should be noted that the estimations under comparison use a different calculation methodology than what

is proposed in this work – the only previous work identified that uses a similar method, yet with slight methodological adaptations in this paper in terms of scope, data sources, and outputs, is Snelson et al. (2024)[21].

To facilitate understanding of the calculation steps and the procedure through output examples, the calculations are accompanied by a summary table , which consolidates all calculation steps and inputs/outputs for the Dutch case over the years. Readers are strongly encouraged to refer to the table for further clarification of the calculations through examples.

Lastly, given the differences in data granularity (country-level versus citizen-enterprise-level), the calculation will involve frequent unit conversions for energy, emissions, and data volume. The conversion calculations will be particularly salient for electricity /emissions–data volume conversions, e.g., kWh-TB; kilograms of CO2e-TB; GWh–EB; or kt. CO_2eq- EB.

Methodology and Calculation Breakdown

STEP 1 - Calculating Total Electricity Consumption and Corresponding GHG Emissions from DC and TN

The initial step is to estimate the total electricity consumption and carbon emissions of data centers (DCs) and telecommunication networks (TNs) for a specific country and year. This part relies on country-level averages of electricity consumption and emissions data officially provided by government bodies (or reports commissioned by them) between 2017 and 2022. This preliminary calculation is necessary because cloud electricity consumption cannot be obtained directly; instead, the aggregate electricity consumption must be estimated to determine the share attributable to cloud services.

The Dutch statistical body (CBS) is the primary data source for total DC electricity consumption from 2017 (1650 GWh) to 2021 (3730 GWh)[59]. The data center's electricity consumption has grown at a relatively stable rate from 2018 to 2021, with an annual growth rate of 15.9% to 17%. Primary data on electricity consumption in Dutch data centers for 2022 were unavailable. However, this value was estimated to be 4320 GWh, assuming an average demand growth rate observed over the five years. More specifically, this work assumes an average growth rate of 16.4% in data center electricity demand in the year 2022 relative to 2021 for the Netherlands only, and exclusively only for the Dutch data center electricity consumption. Both Germany[56,57] and Ireland[60] have direct estimations of DC electricity consumption for the entire period 2017-2022, and therefore no average growth assumption was carried out.

Primary government data about electricity consumption of telecommunication networks at the country level were, on the other hand, not provided by any of the three government bodies. Instead, the TN electricity demand estimation was based on the methodology proposed by TAB[108]: the TN electricity consumption is estimated using operational information (energy intensity of telecommunication networks) retrieved from the integrated annual reports of a primary telecommunication provider (30% or higher of broadband/fixed network national market share) in the country—KPN for the Netherlands[69-76], Deutsche Telekom for Germany[61-68]; and Virgin Media for Ireland[77-80]. In most cases, the data provided by the companies estimates the total electricity consumption (GWh) for telecommunication networks. Based on the market share of the telecommunication provider in its national market (i.e., excluding participation in foreign markets), the market share is extrapolated to the country's national electricity consumption for telecommunication networks, assuming that other telecommunication providers in the country operate systems of similar energy efficiency.

For calculations in the Dutch case, this entails extrapolating KPN's energy consumption (on telecommunication networks) and its respective market share in the Netherlands as a proxy for national electricity consumption. The energy intensity EI_{TN} (kWh/TB) is calculated by dividing the total electricity consumption estimation of the telecommunication company P_i (kWh) for the estimated amount of data volume DV (EB) circulated in the Netherlands through KPN in 2017.

The first step in assessing the EI_{TN} is to estimate the total data volume in the Netherlands and narrow it down to estimates specific to KPN. Unlike Germany and Ireland, the Dutch government provides only partial data on the total data volume in the Netherlands, as it includes only the total mobile data traffic volume. The ITU database uses the same data[41]. Therefore, it is first necessary to estimate the total Dutch data volume, after which to estimate how much of it may be transmitted by the telecom operator. Since the total data volume comprises mobile and fixed networks, the data traffic volume for the latter had to be estimated. To calculate the national volume of the Dutch fixed network, the work adopted the growth rate estimations proposed in Dialogic (2016)[96] and van der Vorst (2018)[97], estimating an average of 40% yearly growth in total data volume traffic (fixed + mobile) in the Netherlands from 2016 to 2022 each year, using 2016 as the baseline year. To estimate the Dutch fixed network data traffic volume, the mobile data traffic volume is subtracted annually from the estimated total data traffic volume for that year.

$$DV_f = DV - DV_m$$

DV_f	Fixed Network Data Volume (PB)
DV	Total data volume (PB)[87-89]
DV_m	Mobile data volume (PB)[87]

Regarding the plausibility of the estimate, the forecast data volume follows the growth rate estimates proposed by Dialogic (2016)[96] and van der Vorst (2018)[97]; for example, the total data volume in 2017 was 140% of the 2016 value. In 2017, this would amount to 3.56 $*10^6$ (TB) of data circulated in the Netherlands. To measure the amount that passes through KPN. The total data volume is multiplied by KPN's share (%) of the Dutch telecommunications market in 2017. With the electricity demand[57,68-75] and the total volume in the territory of the Netherlands[95-97,] it is possible to estimate the energy intensity ratio for telecommunication networks in the Netherlands for 2017:

$$EI_{iTN} = \frac{p_i}{DV_i}$$

$EI_{TN/DC}$	Energy Intensity Ratio of country *i*: (kWh/TB): Telecommunication Networks (TN); OR Data Centers (DC)
p_i	Electricity consumption in country *i* for top network provider (kWh) [56-80]
DV	Total data volume of top network provider in country *i* (TB)[87-98]

This resulted in approximately 220 kWh spent per terabyte (TB) of data in Dutch telecommunication networks in 2017. This ratio is assumed to reflect the operational conditions of TN nationwide. The EI_{TN} ratio is then multiplied by the total amount of data volume circulated in the country for that year to get the electricity consumption (P):

$$P_{TN} = 220\ kWh/TB\ *\ 3.56\ (*\ 10^6)$$

$P_{iTN/DC}$	Electricity consumption in country *i* in a specific year (GWh): Telecommunication Networks (TN); OR Data Centers (DC)

An important differentiation concerns the definition of ‘energy use’: while in Life Cycle Assessment (LCA) design, electricity distribution losses between the point of production and consumption would be allocated as part of the use and, therefore, be included in the emission factor calculation[109], this work applies the carbon accounting practices (e.g. SBTi, GHG Protocol) defining energy use only as its consumption – with such losses being classified as part of the upstream indirect emissions[110,111]. Similar calculations were carried out for each country and year, using the same methodology, for both DC and TN.

Year	DC Electricity Consumption in the Netherlands	TN Electricity Consumption in the Netherlands
2017	1650 GWh	1620 GWh

From this, 1652 GWh arose from data processing and storage (DC) and 1621 GWh from data transmission (TN) across the entire sector. A plausibility check was conducted on TN electricity consumption by comparing the results with those from other studies.

According to Lunden et al.'s (2022)[14] estimates, 1 to 1.5% of electricity consumption can be attributed to telecommunications networks. Ecorys (2023)[27] presents a similar range, estimating TN electricity consumption at 1% of Dutch grid electricity demand. The same estimation range is observed in Kamiya and Bertoldi (2024)[17], who estimate the average EU TN electricity demand at around 1-1.2% in 2022. Considering the share of estimated TN consumption in total electricity consumption in 2017, this work estimates that TN networks required around 1.5-2% of the total electricity consumed in the Netherlands in 2017, within the assumption ranges of previous studies. The same energy intensity ratio is calculated for data centers: EIDC = p/DV for each country and year. However, unlike telecommunication networks, countries already estimate the national electricity consumption of data centers (P_{iDC}).

Once the DC and TN electricity demands have been estimated, the next step is to calculate the associated electricity consumption emissions. Two primary methods for calculating Scope 2 emissions exist in the literature: market-based and location-based. Market-based emissions factors are derived from contractual instruments in each electricity supply contract, which may incorporate additional attributes beyond the amount of GHG emitted by the energy supplier powering the data center, e.g., certificate instruments such as RECs[7]. Conversely, location-based calculations derive emission factors from the average electricity grid location from which the electricity is supplied [25]. Under a market-based method, an emission factor may be reduced compared to its location-based counterpart: the energy contractor may reduce its emissions by contracting energy attributes from renewable sources of the electricity supplier[7]. Yet, although emissions are reduced on an individual (energy supply) basis when aggregating at the level of all data centers and telecommunication networks, there may be unaccounted emissions, e.g., fossil fuel power plants to supply the renewable plant in times of peak demand or for power dispatch services, that are not covered under a market-based method.

For electricity consumption emissions (Es2) (Scope 2 GHG emissions under the GHG Protocol), the work applied a strict Scope 2 location-based method – which, despite sharing a similar name, does not refer to the location-based allocation of emissions. Using the Scope 2 location-based introduced estimation divergences in emissions, compared with some previous studies, for the plausibility check. A significant part of the literature adopts ITU's LCA

assessment methodology[109], allowing for the use of the market-based method for Scope 2 assessment. Using the market-based method would allow the inclusion of energy attributes from suppliers that decrease emission intensity. For example, a market-based Scope 2 emissions approach would entail that 88% of the Dutch DC market operates on renewable electricity[31] and 100% of the Dutch TN[69-76].

Nonetheless, such attributes are compensatory instruments that abate emissions in the inventory but do not reduce them. A market-based methodology could hint that the Dutch DC and TN offset all their emissions and, therefore, emit barely Scope 2 greenhouse gases[15], which is not verifiable. Therefore, this work adopts a location-based assessment for its Scope 2 emissions. Scope 2 emissions are calculated as:

$$E_{s2} = \sum_i p_i \, * \, ef_i \, * \, GWP$$

E_{s2i}	Electricity Consumption Emissions (kt CO_2eq): Telecommunication Networks (TN); OR Data Centers (DC) in country i in a specific year
P_i	electricity consumption of DCs and TNs in country i (kWh) [56-80]
ef_i	the emission factor of average grid electricity consumption in country i (kg CO_2eq/kWh)[81-83]
GWP	Global Warming Potential (*for CO_2 = 1)

The emission factor refers to the amount of CO_2eq per average grid electricity consumption (kWh). The data used was based on primary official government sources issued by the competent authorities for each country and year. After completing this step, there is enough data to estimate the Dutch electricity consumption emissions of DC and TN:

Year	DC Electricity Consumption GHG Emissions in the Netherlands	TN Electricity Consumption GHG Emissions in the Netherlands
2017	743.4 kt CO_2eq	729 kt CO_2eq

Similar to the case of electricity consumption for data centers and telecommunication networks, the emission intensity ratios have also been calculated for both DCs and TNs in all three countries for each year. Given the magnitude differences, where national emissions tend to be of much larger magnitude (due to total data volume in EB), the emission intensity ratio Ei_{s2} uses units more suitable for data volume units at the terabyte (TB) scale (kg CO_2eq):

$$EI_{s2(TN/DC)} = \frac{E_{is2}}{DV_i}$$

$EI_{s2(TN/DC)}$	Emission Intensity Ratio (kg CO_2e/TB): Telecommunication Networks (TN); OR Data Centers (DC) in country i in a specific year
E_{s2}	Electricity consumption emissions of country i (kg CO_2eq)
DV	Total data volume of country i (PB)[87-98]

The calculations thus far cover estimates of the annual environmental impacts of the DC and TNs on electricity and GHG emissions. The following stages will require isolating the extent to which this total impact can be attributed to cloud computing services. This will entail carrying out two significant separations: (a) how much of the total DC and TN impact is related to cloud services, and (b) how much of this cloud impact is imported/exported services. Given the limited availability of data on cloud impact at the national level, the calculation in the next section will involve a change in the level of analysis granularity: from country-level to enterprise and citizen-level.

Since most data on cloud services are available at the citizen- or employee-level, the next part will require isolating the average annual cloud-intensity use (in data volume) for a citizen or employee in the country for that specific year. Once average use intensity is estimated at the enterprise and citizen levels, this data will be used to estimate cloud service use at the country level.

STEP 2- Calculating Cloud Electricity Consumption and Corresponding Emissions to Citizen/Enterprise Level

Calculating the cloud footprint will first require estimating the cloud data volume for citizens and employees. As discussed in the methodology, this work isolates cloud computing at the citizens' and employees' levels because estimating the impact of all cloud use purposes (for citizens' or employees' use) at the country level uniformly would be subject to several uncertainties. In particular, it would imply assuming that cloud service consumption patterns are similar between personal and professional use, which, however, is highly uncertain, given the different types of cloud consumption patterns between personal (e.g., video streaming) and professional (e.g., coding, email).

To isolate the employee and citizen cloud-use intensity, this work applied a variation of the commercial cloud method proposed by Snelson et al. (2024)[21] to separate cloud computing from the total ICT sector. Measuring the share of cloud computing requires assessing the intensity of cloud use. The cloud use intensity requires (i) measuring the range of ICT users adopting cloud computing and (ii) the volume of cloud use among these cloud users vis-a-vis the other uses of data volume to estimate the share of DC and TN electricity consumption that can be assigned to cloud services, specifically. To understand the total cloud impact, these two calculations (i) and (ii) are separated into two dimensions of cloud consumption: citizen (end-user) cloud consumption and enterprise (employee) cloud consumption. The separation stems from the differences in data volume intensity between personal and professional cloud use.

Differentiating cloud use from the rest of ICT use is prone to methodological challenges and lacks a unified approach. The literature presents a wide range of estimation approaches to estimate the share of cloud computing over the total data consumption, incorporating bottom-up to top-down calculation assumptions:

Top-down calculation assumptions: Estimation is built based on macro-level statistics issued by the government or industries, where:

- The consumption of cloud computing is the data on the proportion of cloud workloads in all data centers[41]

Bottom-up calculation assumptions: Estimation is built based on measuring and monitoring operating conditions data at the source, where:

- The consumption of cloud computing is that of hyperscale data centers plus other servers in other data centers dedicated to cloud services[4]

The different assumptions have significant effects on the final estimates of total electricity consumption: results range from an estimated 80% or more of data center electricity consumption attributed to cloud services (top-down) to 30% of data center electricity consumption (bottom-up). As discussed in the main text, the approach used here is based on Snelson et al. (2024)[21] by estimating:

- In a bottom-up approach, the energy consumption of enterprise cloud computing is estimated based on the aggregate of the average cloud data use intensity by a citizen or employee in a given country and year.

The choice of this method stems from its ability to provide higher levels of differentiation for comparative purposes, e.g., between citizen and employee cloud service users and between countries. To exemplify, using an average of the shared IT workload off-site to measure cloud computing would entail using a yearly regional average for all European countries (top-down approach). Applying a bottom-up approach, on the other hand, national data on cloud adoption pinpoints significant differences: in 2023, Germany's cloud adoption among enterprises stands at 47%; yet in Ireland and the Netherlands, for the same year, cloud adoption is above 60%[33].

Cloud Adoption on the citizen level

The goal is to estimate the total data-use intensity (cloud) of citizens using cloud services for each country and year, henceforth labeled CU_I (TB/citizen). However, to calculate CU_I, it is necessary to estimate first the total citizen data volume DV_I (EB), including cloud and non-cloud, used by citizens, divided by the number of citizens using the internet for a specific country and year.

The first step entails estimating the total data volume used by citizens (PB), including cloud and other services – henceforth called DV_I. The first stage is to identify the total citizen data volume (cloud and other) from the entire national data volume, as reported by Cisco Internet. The share of citizen (non-enterprise) data volume is circa 84% of the total Internet data traffic volume yearly between 2017 and 2023[40]. Since data were unavailable for each case, all three countries are assumed to have the same average enterprise data traffic volume (cloud and other) as proposed by Cisco. Using the total data volume already available by governments[87-98] and used for calculating the TN electricity consumption P_{TN}, it is possible to estimate the citizen data volume for a country and year:

$$DV_I = DV * DV_c$$

DV_I	Citizen Data Volume in country i in a specific year (PB)
DV	Total data volume of country i (PB)[87-98]
DV_c	Internet data volume share of citizen use (84%)[40]

In the Netherlands, the citizen traffic volume is estimated to have reached 620 petabytes (PB) in 2017. The number of (individual) cloud users and the total traffic data volume allow the estimation of the data use intensity of citizens in a given country and specific year.

After estimating the citizen data volume, this value is divided by the total number of citizens in the country with internet access using cloud for that specific year, labeled as U_c. This calculation used Eurostat country data about the total population multiplied by (a) the share percentage of households with internet access, times (b) the percentage share of households with internet access that use cloud services for each country and year[38,39,100]. In 2017, in the Netherlands, there were 17 million and 80 thousand citizens, of whom 98.2% had internet access, accounting for 16 million and 770 thousand Dutch citizens with internet access. Out of this total, 48.84% had cloud use:

$$U_C = c * H_i * c_{ar}$$

U_c	Number of Citizens using Cloud in country i in a specific year
c	Number of citizens in country i [39]
H_I	Households with internet access in country i (% adoption)[38]
c_{ar}	Cloud adoption rate (individual use) (% adoption)[101]

This general data intensity measures how much data an individual uses in the country in a specific year:

$$DUI = \frac{DV_I}{c * H_i}$$

DUI	Data Use Intensity (Citizen Use) in country i in a specific year (TB/ citizen)

DV_I	Citizen data volume (TB) in country i
$c * H_i$	Individual internet users in country i (number of households w/internet access * number of citizens)

In 2017, the intensity of data use in the Netherlands for citizens was estimated to be 0.36 terabytes (TB) per citizen with internet access. This ratio only refers to personal use and excludes professional use – which will follow a different calculation process described later in this section. With the data use intensity ratio, it is now possible to infer the total amount of cloud data used by individual consumers:

$$DVT_I = DUI * U_C$$

DVT_I	Total Data Use by Citizens (that use cloud and other services) in country i in a specific year (PB)
DUI	Data use intensity (citizen use) (TB/citizen user)
U_c	Individual cloud users (citizen)

The $DVT_{I.}$ assumes uniform data use in the country, i.e., citizens who use cloud services use as much data as those who don't use cloud services (but use the Internet).

In the Netherlands, total data use by citizens (using cloud and other services) amounted to approximately 3024 petabytes (PB) in 2017. Given the overall citizen data volume, it is necessary to estimate how much of the total volume is attributable to cloud computing. The calculation assumed the yearly IT workload distribution forecast from the Uptime Institute Data Center Survey. Given the absence of data sources indicating personal preferences of cloud use, this research assumed similar preferences of cloud use for personal and professional use, based on Uptime Institute data. In 2017, Uptime estimated that 40% of an enterprise IT workload is off premises, i.e., cloud[42,43], and the same share was assumed for the citizen-level:

$$CU_I = DVT_I * o$$

CU_I	Cloud Data Use by Citizens in country i in a specific year (TB)
DVT_I	Total data use by citizens (TB)
o	Off-Site IT workload rate (%)[42,43]

In 2017, the Netherlands used approximately 130 petabytes (PB) of cloud data for individual consumption. The calculation allowed an estimation of the volume from individual cloud consumption in a non-commercial (personal) setting.

Cloud adoption on the employee-level

The goal is to estimate the total data-use intensity (cloud) of employees using cloud services for each country and year, henceforth labeled CU_E (TB/employee). However, for calculating CU_E, it is necessary to estimate first the total employee data volume DV_E (PB), including cloud and non-cloud, used by employees, divided by the number of employees using cloud for each country and specific year.

The first stage is to identify the total employee data volume (including cloud and other services), which is used as a basis for the average provided by the Cisco Internet reports – the share of enterprise data volume is circa 16% of the total internet data traffic volume yearly between 2017 and 2023[40]. Given that data were unavailable for each specific case but only at the international average level, like for citizens, all three countries are assumed to share the same average total enterprise data traffic volume as reported by Cisco. Using the total traffic volume already available by governments (which was also used to calculate the TN electricity demand), it is possible to estimate the enterprise data traffic volume for a specific country and year:

$$DV_E = DV_T - DV_I$$

DV_E	Employee Data Volume in country i in a specific year (TB)
DV	Total data volume (PB)[87-98]
DV_I	Citizen data volume (PB)

In the Netherlands, the enterprise traffic volume is estimated to have reached 1800 petabytes (PB). in 2017. The number of enterprise employees and the total traffic data volume allow estimation of the data use intensity of employees in a given country and year. This general data intensity measures how much data an employee uses in a country and year. However, since the Cisco data only refers to enterprise data and not to public administration employees, it is first necessary to measure the number of enterprise employees, specifically:

After estimating the total volume, the next step is to estimate the number of employees using the cloud. Calculating the number of employees using cloud follows a similar intensity-based approach to that applied at the citizen level, but with an additional distinction between public and private employees. This difference is because private enterprises almost exclusively use cloud data, and because cloud service use in the civil service is significantly less documented. Therefore, we perform calculations only for private enterprises and assume the public sector has a similar use pattern. The calculation includes finding the total number of employees using cloud services for each country and year. This was done using Eurostat and country data about cloud adoption among enterprises for each country and year[33], and country employment data. In 2017, in the Netherlands, cloud services were adopted by approximately 41.3% of enterprises[33], and it is assumed that all employees of these companies apply cloud services. As mentioned in the methodology, it is assumed for the calculation that the cloud has a similar adoption rate between the public and private sectors:

$$U_E = e * c_{re}$$

U_E	Number of Employees using Cloud (Enterprise Employee Cloud Users) in country i in a specific year (employee)
e	Number of employees in country i (enterprise and public) [34,38,99]
c_{re}	Cloud adoption rate (enterprise use) (%)[33]

It is estimated that approximately 3.3 million of the Netherlands' 8.1 million (private enterprise) employees had access to or used cloud services in 2017. The number of enterprise employees and the total traffic data volume allow the estimation of the data use intensity of employees from a given country and year. This general data intensity measures how much data an employee uses in the country and year. However, since the Cisco data only refers to enterprise data and not to public administration employees, it is first necessary to measure the number of enterprise employees, specifically:

$$E_e = e - e_{pa}$$

E_e	Number of Enterprise Employees in country i in a specific year (employee)
e	Total number of employees in country i (enterprise and public) [34,38,99]
e_{pa}	Total number of public administration employees in country i [34,38,99]

$$DUI_E = \frac{DV_E}{E_e}$$

DUI_E	Data Use Intensity (Employee Use) in country i in a specific year (TB/employee)
DV_E	Employee data volume (TB)
E_e	Number of enterprise employees in country i

In 2017, the Dutch data use intensity was estimated at 0,15 terabytes (TB) of data per employee. This ratio refers only to use as an individual in the employee condition, i.e., performing company duties, excluding personal use (e.g., video

streaming). Now, the assumption about the same patterns of cloud use between enterprise and public administration is applied to estimate the total data use by employees:

$$DVT_E = DUI_E * e$$

DVT_E	Total Data Use by Employees (that use cloud and other services) in country *i* in a specific year (PB)
DUI_E	Data use intensity (employee use) (TB/employee) in country *i*
e	Number of employees in country *i* (enterprise + public)

In the Netherlands, this total data amounted to approximately 519 petabytes (PB) in 2017. Lastly, given the enterprise-wide and public employee data volume, it is necessary to determine how much of the total volume is attributable to cloud computing. To separate the volume, this work adopted the same workload distribution (on-site/off-site) as provided by the annual Uptime Institute Data Center Survey. In 2017, Uptime estimated that 40% of an enterprise IT workload is off-premises[42,43]. As mentioned, these estimates are from enterprise workloads and were used for citizens due to the scarcity of projections on personal cloud use. The total cloud use by employees (CU_E) is calculated as:

$$CU_E = DVT_E * o$$

CU_E	Cloud Data Use by Employees in country *i* in a specific year (PB)
DVT_E	Total data use by employees (that use cloud and other services) (TB) in country *i*
o	Off-Site IT Workload Rate[42,43] (%)

In 2017, the Netherlands used 223 petabytes (PB) of cloud data. After completing these calculations, the citizen-and-enterprise (public and enterprise employees) level cloud data volume was isolated and used to estimate the country level.

Cloud adoption at the national level

The next step entails estimating the national volume of cloud computing data for each country and year. This estimation was made with consideration of the inherent limitations in the Eurostat data description. This limitation in calculating energy and emissions concerns the Eurostat data description of how cloud adoption is counted across the household and enterprise-level datasets. According to the available descriptions of the datasets, it is unclear whether the interaction between enterprise and household cloud adoption data points is separate, i.e., the household adoption dataset excludes any observations from the enterprise use dataset, or together, i.e., the household adoption dataset incorporates the enterprise use dataset. In the latter case, this uncertainty raises the risk of double-counting: the same cloud use might be counted twice – once for personal use and again as an employee. Considering this uncertainty, the calculation of cloud computing data volume used a range assumption for estimating the total cloud data volume:

- Upper Range: When it is assumed that citizen and employee cloud adoption dataset points are fully separated, i.e., no risk for double counting, then the calculation of the data volume (TB) from the total cloud computing (citizen and enterprise) for the upper range goes as:

$$CU_{UR} = CU_I + CU_E$$

- Lower Range: When it is assumed that citizen and employee cloud use overlap, i.e., the citizen adoption also includes employee adoption. The calculation of the data volume (TB) from the total cloud computing for the lower range goes as:

$$CU_{LR} = CU_I - CU_E$$

- Middle Range: Given the boundaries, the middle range estimate used throughout the manuscript main 'Results' section uses the average (mean) value between the range boundaries, while Upper and Lower boundaries are presented in greater detail in Document S2. The calculation of the data volume from the total cloud computing for the middle range goes as:

$$CU_{MR} = \frac{CU_I + CU_E}{2}$$

CU_{UR}	Cloud Upper Range Volume in country i in a specific year (PB)
CU_{LR}	Cloud Lower Range Volume in country i in a specific year (PB)
CU_{MR}	Cloud Middle Range Volume in country i in a specific year (PB)
CU_I	Cloud data used by citizens in country i (TB)
CU_E	Cloud data used by employees in country i (TB)

At this stage, since the cloud data volume estimates are levelized nationally, it is now possible to estimate the average electricity consumption and corresponding emissions of cloud computing across DC and TN, as described below.

Nonetheless, before proceeding to the next step, some clarification on data intensity is required to understand why this method was adopted. First, alternative methods to the intensity metrics used (TB/employee or citizen) acknowledge and better handle the hidden complexities of data transmission for estimating outputs[20], but they also impose a higher implementation threshold for comparative analysis. Based on data use types, most countries lack aggregate data on all TN and DC data use activities occurring within their territory. Although energy intensity or carbon intensity ratios may yield less accurate future estimates, they offer greater normalization value and, consequently, greater comparative or reproducibility potential for most countries worldwide, which often lack the data depth of European nations.

Second, the hidden complexities for the next calculation steps, specifically when estimating cross-border flows of cloud computing services. The greater accuracy of the newer estimation methods stems from the greater differentiation in the energy consumption of distinct computational tasks, allowing more precise measurement[19,20]. The task differentiation would impose a significant complexity threshold for the case of cloud cross-border data volume: it would require tracing not only cross-border data's foreign origin/destination but also the flow, i.e., how much comes from where and from which computational task: emails, video streaming, machine learning, and others. The differences have significant effects on electricity consumption, e.g., the average electricity consumption of a typical Google search is estimated at 0.3Wh, whereas OpenAI's ChatGPT is estimated at 2.9Wh per request[103]. Albeit desirable, the complexity of this data depth exceeds the available data, particularly considering that such methods are being developed and this work uses historical data.

STEP 3- Calculating the Total Electricity Consumption and Corresponding GHG Emissions from Cloud Computing

Since the boundary parameters for cloud use (including both citizens and employees) are set, it is possible to calculate the service's footprint over the DC and TN for each country and year. The total electricity consumption and

GHG emissions of cloud computing as a whole and the cross-border data flow are estimated by multiplying the total cloud data amount by the energy and emission intensity ratios:

$$PC_{i,j} = \sum_{i} CU_j * EI_{i(TN/DC)}$$

$PC_{i,j}$	Cloud Electricity Consumption of country *i* for cloud data volume range *j* in a specific year (kWh)
CU_j	Cloud data volume (TB): j=UR (upper range), MR (middle range) OR LR (lower range) in country *i* in a specific year
$EI_{i(TN/DC)}$	Total electricity intensity ratio of country *i* (kWh/TB): Telecommunication Networks (TN) / Data Centers (DC) in country *i* in a specific year

$$EC_{is2,j} = \sum_{i} CU_j * EI_{is2(TN/DC)}$$

$EC_{is2,j}$	Cloud Electricity Consumption Emissions of country *i* for cloud data volume range *j* in a specific year (kt CO_2eq)
CU_j	Cloud data volume (TB): j=UR (upper range), MR (middle range) OR LR (lower range) in country *i* in a specific year
$EI_{is2(TN/DC)}$	Total emission intensity (electricity consumption) ratio of country *i* (kg CO_2e/TB): Telecommunication Networks (TN); OR Data Centers (DC) in country *i* in a specific year

To facilitate unit comparison with national levels of cloud electricity consumption and corresponding emissions for the combined DC and TNs, the aggregate is provided at the 10^6-magnitude level relative to the reference unit, along with the energy and carbon-intensity ratios. Concerning electricity consumption, from kWh/TB of energy intensity to GWh for the total cloud electricity consumption; concerning emissions, from kg CO_2eq/TB to kt. CO_2eq for total cloud emissions. The results were also used to calculate the cloud computing share of the DCs and TNs for the given country and year. Using the Dutch example:

Year	Total Cloud Electricity Consumption	Cloud Electricity Share (from the ICT services)	Cloud Electricity Consumption Emissions	Cloud Emissions Share (from the total DC and TN Emissions from Electricity Consumption)
2017	478 GWh - 677 GWh	14.5 – 20.7 %	215 – 300 kt CO_2eq	14.6 – 20.4 %

After separating cloud computing from the total ICT services sector, the last step entails subdividing cloud computing's environmental impact according to the data flow consumption: (a) what is produced and consumed domestically; (b) what is produced domestically but exported; and (c) what is imported from abroad to be consumed domestically.

STEP 4 - Calculating the Electricity Consumption and Corresponding GHG Emissions According to Cloud Flow Patterns

In this study, turnover from international trade was estimated using the method proposed by Hasanbeigi and Darwili (2022)[44] to assess embodied carbon in the international trade of manufacturing goods based on international trade patterns. As mentioned in the methodology, it follows the assumption that:

- The cloud data flow is calculated based on the proportion of the economic value (EUR) from the international trade of ICT services vis-à-vis the national account turnover of the ICT services

The first step entails isolating the turnover share of the cross-border flow of ICT services, including cloud services and other services, (exports + imports) over the total turnover (national accounts) from the ICT services sector for a given country and year:

$$CB_{ES} = \frac{t_I}{t_T}$$

CB_{ES}	ICT Services Economic Significance According to Destination (Domestic / Export / Import) in country *i* in a specific year (%)
t_I	Total international ICT services trade turnover (cloud and other services) in country *i* in a specific year (billion EUR)[101-105]
t_T	Total ICT services turnover (cloud and other services) (billion EUR) in country *i* in a specific year[101-105]

In the Netherlands, international data flows are estimated to have generated almost 50% of the ICT services sector's total turnover, of which 30% stems from exported ICT services and 20% from ICT services imported into the country. For the analysis of cloud services, specifically, it is assumed that cloud computing data volume (PB) follows the same value-generation share of ICT services as a whole. If 20% of the ICT value is exported, it is estimated that 20% of the cloud data volume is for exports. This assumption rests on the fact that, given the inherent uncertainties surrounding cross-border data flows (origin/destination of data volumes), the relative economic importance of ICT is used as a proxy for estimating data volume flows. Additional details are provided at the end of this section:

$$DV_{CB} = CB_{ES} * CU_j$$

DV_{CB}	Cloud Data Volume per Category (Domestic /Export / Import) in country *i* in a specific year (TB)
CB_{ES}	ICT services economic significance according to destination (Domestic / Export / Import) in country *i* in a specific year (% of total turnover)
CU_j	Cloud data volume (TB): j=UR (upper range), MR (middle range) OR LR (lower range) in country *i* in a specific year

Among the 50% (534- 645 PB) turnover of cross-border cloud services in the Netherlands in 2017, 30% (322 -389 PB) of their total value was generated from exporting ICT services, and 20% (212 – 256 PB) from imports, providing a net balance of 10% (110 -133 PB) from cloud data outflow in the form of ICT services.

Before proceeding to the estimates, it should be noted that there is an important caveat regarding the differences between hosting the cloud domestically (domestic and export cloud scenarios) and hosting the service overseas (import cloud). In import, the cloud is hosted in a data center with different energy consumption and grid mix than those of the domestic/export cloud. Therefore, in a real-life case, the import should be calculated using the electricity consumption/emission factor for the foreign grid where the data center is located. Nevertheless, it is extremely difficult to estimate cloud data volume flows with confidence using the available data. To mitigate this uncertainty, the estimations in the main text assumed a counterfactual scenario for the origin of imported cloud:

- The imported cloud is estimated based on the operating conditions as if that cloud service was hosted domestically

In practice, the imported cloud is assumed to have the same origin parameters as the domestic and export cloud (same energy and carbon intensities as of hosted domestically). This uncertainty in the estimate is discussed in greater detail in the sensitivity analysis section below.

In the last stage, the total electricity consumption and GHG emissions of cloud computing and the cloud data flow are estimated by multiplying the total cloud data amount by the energy and emission intensity ratios (calculated in STEP 1).

$$PC_{CB} = DV_{CB} * EI_{TN/DC}$$

PC_{CB}	Cloud Electricity Consumption per Category (Domestic /Export / Import) in country i in a specific year (GWh)
DV_{CB}	Cloud data volume per destination category (domestic /export / import) in country i in a specific year (TB)
$EI_{TN/DC}$	Electricity intensity ratio of country i (kWh/TB): Telecommunication Networks (TN) / Data Centers (DC) in country i in a specific year

$$EC_{CBs2} = DV_{CB} * EI_{s2(TN/DC)}$$

EC_{CBs2}	Cloud Electricity Consumption Emissions per Category (Domestic /Export / Import) in country i in a specific year (kt CO_2eq)
DV_{CB}	Cloud data volume per destination category (domestic /export / import) in country i in a specific year (PB)
$EI_{s2i(TN/DC)}$	Emission Intensity Ratio: Telecommunication Networks (TN); OR Data Centers (DC)

The calculations provide the following output for the Netherlands in 2017 about the electricity consumption and emissions of cross-border cloud computing in data centers:

Impact Type (Cross-Border)	Total Cloud	Export	Import	Net Balance
DC- Electricity Consumption	119 - 169 GWh	72 - 102 GWh	47 - 67 GWh	25 - 35 GWh
DC – Electricity Consumption GHG Emissions	54 - 76 kt CO_2e	32 - 46 kt CO_2e	21 - 30 Kt.CO_2e	11 - 16 kt CO_2e

And for telecommunication networks:

Impact Type	Total Cloud	Export	Import	Net Balance
TN- Electricity Consumption	117 - 166 GWh	71- 100 GWh	46 - 66 GWh	24 – 34 GWh
TN – Electricity Consumption GHG Emissions	38 - 76 kt CO_2e	23 - 52 kt CO_2e	15 -34 kt CO_2e	8 -18 kt CO_2e

Interpreting the estimates entails that, in 2017, cloud computing consumed between 478 to 677 GWh from the Dutch national grid 2017, out of which between 236 to 335 GWh are from cross-border cloud services. Out of the cross-border total, approximately 143 to 202 GWh were to supply services used overseas, 93 to 133 GWh were imported from cloud services hosted overseas. Converting to electricity consumption and carbon emissions using the national grid average, the total Dutch net balance of cross-border data flow pinpoints an additional 19-34 kt CO2 equivalent of emissions added to the Dutch emission inventory from overseas consumption of cloud applications hosted in the Netherlands. Therefore, in 2017, the Netherlands was a net carbon exporter of cloud computing services, accounting for around 27 kt of emissions in its emission inventory that was consumed elsewhere.

One last caveat lies in why this work uses the turnover of ICT services as a proxy for measuring the magnitude of different consumption types of cloud data flow (domestic/import/export). Other methodologies were considered but not applied to estimate cross-border flow. Notably, a discarded methodology estimated the profitability ratio of data volume (terabytes of data required to generate a million euros). Such an estimation process assumes that cloud services operate on a proportionate ratio of economic value addition, which is not verifiable. Cloud computing encompasses a wide range of services, among which some have a direct link between data use and added economic value (e.g., machine learning), but for several other cloud services, this link is indirect (e.g., sending/receiving emails, video streaming). To illustrate the problem, using the profitability ratio (TB/million euros) for the three countries yields 395 TB/million euros for Germany, 621 TB/million euros for the Netherlands, and 7.3 TB/million euros for Ireland in ICT services in 2022. Interpreting these outputs literally would suggest that Ireland is almost 100 times more efficient in generating economic value from ICT services than the Netherlands, which is not verifiable by empirical data[113]. Instead, it is verified that Ireland's headquarters are large-scale IT companies focused on high-end ICT service[114,115] but the country has average European-level digitalization rates[33], allowing it to generate high economic value with a significantly lower amount of data. Conversely, due to its higher digitalization rate, the Netherlands uses digital services more intensively and has adopted them for a wider array of consumption purposes (e.g., email and video streaming), many of which do not add direct economic value. Thus, on average, more Dutch data is required to add economic value. The table below summarizes the calculation process for the Netherlands:

Table S2. Input parameters and calculated parameters for the case of the Netherlands

**Parameter [unit]**	**Parameter description**	**Values applied**					
		**2017**	**2018**	**2019**	**2020**	**2021**	**2022**
p_i [GWh]	Total data center (DC) electricity consumption in country i in a specific year	1652	2367	2747	3184	3730	4327
	Total telecommunication network (TN) electricity consumption in country i in a specific year	1621	1973	2033	1847	2238	2336
ef_i [kg CO2eq/kWh]	Emission factor of average grid electricity consumption in country i in a specific year	0.45	0.43	0.37	0.29	0.30	0.27
GWP	Global Warming Potential for CO_2	1	1	1	1	1	1
DV [PB]	Total data volume (mobile + fixed) in country i in a specific year	7370	14510	20450	28900	40940	58980
EI_i [kWh/TB]	Total electricity intensity of DCs in country i in a specific year	224	163	134	110	91	73
	Total electricity intensity of TNs in country i in a specific year	79	74	100	49	54	28
E_{is2} [kt. CO_2eq]	Total DC electricity consumption GHG emissions in country i in a specific year	743	1018	1016	923	1119	1168

	Total TN electricity consumption GHG emissions in country i in a specific year	729	848	752	536	671	631
EI_{s2i} [$kgCO_2eq/TB$]	Total emission intensity (electricity consumption) of DCs in country i in a specific year	319	275	218	152	189	215
	Total emission intensity (electricity consumption) of TNs in country i in a specific year	35	29	34	15	19	9
DV [PB]	Total data volume (mobile + fixed) in country i in a specific year	7370	14510	20450	28900	40940	58980
DV_c [%]	Internet data volume share of citizen use	84	84	84	84	84	84
c	Total number of citizens in country i (millions)	17.08	17.18	17.28	17.40	17.47	17.59
H_I [%]	Households share internet access in country i	98.23	98	98.41	96.95	98.56	98.28
c_{ar} [%]	Cloud adoption rate in country i (individual use)	48.84	47.82	52.23	47.65	52.3	57.42
o [%]	Off-Site IT workload rate	43	40	41	42	46	52
e	Total number of employees in country i (enterprise + public) (millions)	9.15	9.20	9.22	9.24	9.28	9.30
c_{re} [%]	Cloud adoption rate in country i (enterprise use)	41.3	48.2	50.4	52.6	64.9	63.1
e_{pa}	Total number of public administration employees in country i (millions)	0.50	0.51	0.52	0.53	0.55	0.57
DV_I [PB]	Total citizen data volume	6190	12190	17180	24280	34390	49550
U_c	Total number of citizens using cloud in country i (millions)	8.19	8.05	8.88	8.04	9	9.92
DUI [TB/ citizen]	Data use intensity in country i (citizen)	0.36	0.72	1.01	1.43	1.99	2.86
DVT_I [PB]	Total data use by citizens (that use cloud and other services)	3020	5820	8970	11560	17990	28450
CU_I [PB]	Total cloud data use by citizens in country i	1300	2330	3670	4850	8990	13080
DV_E [PB]	Total employee data volume in country i	1180	2320	3270	4620	6550	9440
U_E	Total number of employees using cloud in country i (millions)	3.78	4.44	4.64	4.86	6.02	5.8
E_e	Number of enterprise employees in country i	7.59	7.81	7.97	7.85	8	8.29
DUI_E [TB/ employee]	Data use intensity in country i (employee)	0.15	0.29	0.41	0.58	0.81	1.13
DVT_E [PB]	Total data use by employees in country i (that use cloud and other services)	510	1190	1750	2590	4540	6360

CU_E [PB]	Total cloud data use by employees in country *i* in a specific year	220	470	720	1090	2270	2920
CU_{UR} [PB]	Cloud upper range volume in country *i* in a specific year	1520	2800	4390	5950	11270	16010
CU_{LR} [PB]	Cloud lower range volume in country *i* in a specific year	1070	1850	2950	3760	6720	10150
CU_{MR}[PB]	Cloud middle range volume in country *i* in a specific year	1300	2330	3670	4850	8990	13080
CU_j [10^6 TB]	Total cloud volume range (upper / lower/ middle ranges) in country *i* in a specific year	Values provided in Step 2: Calculated parameters [CU_{UR} ; CU_{LR} ; CU_{MR}]					
EI_i [kWh/TB]	Total electricity intensity of (DCs /TNs) in country *i* in a specific year	Values provided in Step 1: Calculated parameters [$EIi - DC$; $EIi - TN$]					
EI_{s2i} [$kgCO_2eq$/TB]	Total emission intensity (electricity consumption) of (DCs/TNs) in country *i* in a specific year	Values provided in Step 1: Calculated parameters [$EI_{s2i} - DC$; $EI_{s2i} - TN$]					
$PC_{i,j}$ [GWh]	Total cloud electricity consumption of country *i* for cloud data volume range *j* in a specific year – (I) Upper range	677	840	1000	1005	1439	1559
	Total cloud electricity consumption of country *i* for cloud data volume range *j* in a specific year – (II) Lower range	478	555	673	636	858	989
$EC_{is2,j}$ [kt CO_2eq]	Total cloud electricity consumption GHG emissions of country *i* for cloud data volume range *j* in a specific year– (I) Upper range	307	394	437	380	616	634
	Total cloud electricity consumption GHG emissions of country *i* for cloud data volume range *j* in a specific year– (I) Lower range	217	260	294	241	367	402
t_I [billions EUR]	Total international ICT services trade turnover (cloud and other services)	37.17	38.46	43.49	50.88	53.45	64.90
t_T [billions EUR]	Total ICT services turnover (cloud and other services)	75.02	81.45	88.61	90.87	91.70	97.22
CU_j [PB]	Cloud volume range (upper / lower/ middle ranges) in country *i* in a specific year	Values provided in Step 2: Calculated parameters [CU_{UR} ; CU_{LR} ; CU_{MR}]					
EI_i [kWh/TB]	Total electricity intensity of (DCs /TNs) in country *i* in a specific year	Values provided in Step 1: Calculated parameters [$EIi - DC$; $EIi - TN$]					
EI_{s2i} [$kgCO_2eq$/TB]	Total emission intensity (electricity consumption) of (DCs/TNs) in country *i* in a specific year	Values provided in Step 1: Calculated parameters [$EI_{s2i} - DC$; $EI_{s2i} - TN$]					

CB_{ES} [% of total turnover]	ICT services economic significance according to data flow –(I) Domestic	50.45	52.78	50.91	44.01	41.71	33.24
	Data flow – (II) Export	29.90	27.86	28.52	35.01	37.75	39.66
	Data flow– (III) Import	19.65	19.36	20.57	20.98	22.54	27.10
DV_{CB} [PB] – Upper Range	Total cloud volume per category – (I) Domestic	750	1480	2240	2610	4700	5320
	Category – (II) Export	450	780	1250	2080	4020	6350
	Category – (III) Import	300	540	900	1240	2540	4340
DV_{CB} [PB] – Lower Range	Total cloud volume per category – (I) Domestic	540	970	1500	1650	2800	3370
	Category – (II) Export	320	510	840	1310	2400	4020
	Category – (III) Import	210	350	600	790	1510	2750
PC_{CB} [GWh] – Upper Range	Total cloud electricity consumption per category– (I) Domestic	341	443	523	456	685	601
	Category– (II) Export	202	234	293	363	587	718
	Category– (III) Import	133	163	212	217	370	490
PC_{CB} [GWh] – Lower Range	Total cloud electricity consumption per destination category– (I) Domestic	241	293	352	289	409	381
	Category– (II) Export	143	155	197	230	350	455
	Category– (III) Import	94	107	142	138	221	311
EC_{CBs2} [kt. CO_2eq] - Upper range	Total cloud electricity consumption GHG emissions per category– (I) Domestic	154	191	194	132	206	162
	Category– (II) Export	91	101	108	105	176	194
	Category– (III) Import	60	70	78	63	111	132
EC_{CBs2} [kt. CO_2eq] - Lower range	Total cloud electricity consumption GHG emissions per category– (I) Domestic	123	136	134	80	123	90
	Category– (II) Export	56	61	71	69	105	131
	Category– (III) Import	37	42	51	41	66	89

Participation of the DC and TNs in the Nationwide Final Metered Electricity Consumption and Participation of Cloud in the Total Data Volume

This document provides the results for the total electricity consumption and corresponding greenhouse gas emissions of the DC and TN in the top three EU DC hub countries of Germany, Ireland, and the Netherlands, over the total electricity demand and corresponding emissions in these countries, respectively. This estimation delineates the range of the maximum magnitude of environmental impacts attributed to cloud computing and the aggregate environmental footprint of DC and TN services. The second part provides additional information on the share of the cloud's data volume in the total data volume, by consumption category (individual or enterprise).

The Table summarizes the amounts and shares of total electricity consumption and corresponding emissions for the DCs and TNs across the three countries between 2017 and 2022. For the total share value of electricity consumption, Germany experienced an accumulated 40% growth in DC and TNs in 2022 compared to 2017, reaching over 5-6% of total electricity demand by 2022. Accordingly, Germany's share of electricity consumption from TNs has increased, reaching almost 2% of total demand by 2020. Ireland experienced the highest growth and share value in total electricity consumption: the DCs and TNs' consumption share in 2022 was approximately 240% of the 2017 share, reaching an aggregate of 19% of total electricity consumption nationwide in that year. Conversely, the Netherlands holds the lowest share value for the same year, at 5% of total electricity demand in 2022, and has seen an 80% increase over the five years compared to 2017.

Moreover, the magnitude of the emission footprint share varies. Germany and the Netherlands' Scope 2 DC and TN emissions together amount 5% of the total national Scope 2 emissions in 2022. Yet, the carbon footprint shares over total Scope 2 emissions is much higher in Ireland, where, aligned with the electricity consumption scale, the Scope 2 GHG emissions share from DC and TN is estimated to be over twice this value (18%) for the same year.

Table S3. Amount and Share of Data Center (DC) and Telecommunication Network (TN) Electricity Consumption and Corresponding Emissions over the Total National Electricity Consumption and Scope 2 Emissions (2017-2022)

	Electricity Consumption (GWh)					
	Germany		Ireland		Netherlands	
Year	DC	TN	DC	TN	DC	TN
2017	13300	5900	1800	200	1700	1600
2018	14000	7800	2200	200	2400	2000
2019	15100	6600	2500	400	2800	1900
2020	16300	9500	3000	300	3200	1700
2021	17000	9600	4000	400	3800	1500
2022	17900	8900	5300	200	4300	1400
	Electricity Consumption Share Over Total Electricity Consumption Share					
Year	DC	TN	DC	TN	DC	TN
2017	3%	1%	7%	1%	1%	1%
2018	3%	2%	8%	1%	2%	2%

2019	3%	1%	9%	1%	2%	2%
2020	3%	2%	11%	1%	3%	2%
2021	3%	2%	14%	1%	3%	1%
2022	4%	2%	18%	1%	4%	1%
	Electricity Consumption Emissions (kt CO_2eq)					
	Germany		Ireland		Netherlands	
Year	DC	TN	DC	TN	DC	TN
2017	6500	2900	800	100	700	1500
2018	6600	3700	800	100	1000	1900
2019	6200	2700	800	100	1000	1700
2020	6000	3500	900	100	900	1400
2021	7000	4000	1400	100	1100	1600
2022	7800	3900	1700	100	1200	1500
	Electricity Consumption Emissions Share Over Total Electricity Consumption Share					
Year	DC	TN	DC	TN	DC	TN
2017	2%	1%	7%	1%	2%	2%
2018	2%	1%	8%	1%	2%	2%
2019	3%	1%	9%	1%	2%	2%
2020	3%	2%	11%	1%	3%	2%
2021	3%	2%	14%	1%	3%	1%
2022	3%	2%	17%	1%	4%	1%

The estimates the total share of data volume arising from cloud computing services, subdividing into two categories: the share from individual cloud consumers (citizens) and the share from employees (employees). Across the three cases for individual share, Germany recorded the largest increase in volume share (107%) over the six years. In contrast, the Netherlands had the lowest share increase (26%), with a growth pattern similar to Ireland's (29%). Despite a lower increase in the share of cloud data volume, the Netherlands has maintained its position as the country with the highest cloud share among individual consumers throughout the period.

Conversely, employee cloud accounts for a relatively small share of total ICT service data volume (ranging from 1% in Germany in 2017 to 6% in the Netherlands in 2021). Still, its relevance gradually increases in all cases. Between the three DC hubs, the Netherlands has kept its position with the highest cloud share, Ireland kept the second highest share throughout the period, with both keeping an accumulated growth at 160% from the 2017 share value, while Germany has had the lowest cloud share, albeit with the highest share growth in the period at 250% of the 2017 value.

	Country					
	Germany		Ireland		Netherlands	
Year	Employee	Citizen	Employee	Citizen	Employee	Citizen
2017	1%	10%	3%	15%	3%	18%
2018	2%	10%	3%	14%	3%	16%
2019	2%	11%	3%	17%	4%	18%
2020	3%	13%	4%	16%	4%	17%
2021	4%	18%	5%	20%	6%	22%
2022	4%	20%	5%	20%	5%	22%

Sensitivity Analysis

Since the yearly emissions factors and electricity consumption data are directly provided by the government bodies, they are assumed to reflect accurate operating conditions for when the cloud is hosted domestically (domestic and export cases). However, even on domestic conditions provided by the government, there is substantial uncertainty about three factors: (a) the uncertainty about double-counting cloud computing adoption for personal and professional use; (b) employee data volume share over the entire Internet total data traffic volume for all three countries, with other sources pointing to values higher than its assumed baseline value[21]; and (c) the share of cloud workload over the total IT enterprise workload, possibly 30% higher depending on the source[115]. Additionally, there are uncertainties about cloud enterprise adoption for Germany, which, although provided directly by Eurostat[33,100], differs substantially (almost 50% lower) from the digitalization levels discussed by the European Commission[117]. To facilitate the understanding of variations, this work used the year 2022 as a sample of variation:

Table S4. Sensitivity Analysis Inputs (2022 reference)

Input	Input Change	Input Value	Input Change Source
Enterprise Internet Data Traffic Volume	+ 44% of employee internet traffic	23% of total internet data volume is from employee use (enterprise)	Snelson et al.[22]
	- 10% of individual internet traffic	77% of total internet data volume is from citizen use (individual)	
Cloud Workload Share Over Total IT workload	+30%	60% total workload	Thales Group[108]
Cloud Enterprise Adoption	+ 47% for Germany	65% of enterprises adopt cloud	European Commission[109]
	+ 3% for Ireland		
	+ 6% for the Netherlands		

Based on the estimated variations, the table illustrates how the input variations above affect the estimated outputs for electricity consumption and corresponding emissions in 2022. The plausible scenario represents a combination of input variations within a likelihood range for net balance estimates, i.e., a range in which an increase in one or more inputs from the table above may increase the magnitude of cross-border cloud computing electricity consumption and the corresponding emissions estimate relative to the baseline value. Each country has a different plausibility scenario because of its distinct shares of cross-border cloud computing flow over total cloud computing and the ICT services– the higher the share of cross-border cloud flows, the higher the magnifying impacts and, thus, the estimation skewing risks. The scenario limit marks unlikely, skewed values, representing the net balance output when all inputs are set to their highest values relative to their benchmarks. The table below summarizes the estimates of the outputs for electricity consumption and emissions:

Table S5. Output Estimation of Total GHG Emissions from Electricity Consumption (2022) (% of Baseline Value for the Range)

	Country					
	Germany		Ireland		Netherlands	
Scenario	Upper Range	Lower Range	Upper Range	Lower Range	Upper Range	Lower Range
Plausible Scenario	+ 30%	-35%	+80%	+35%	+10%	-55%
Scenario Limit	+150%	-15%	+ 130%	+75%	+130%	-45%
Average Output Variation Range per Country (% Upper / % Lower)						
% Range from Baseline Scenario	+185%		+33%		+120%	

Both Germany and the Netherlands tend to present estimation variations in which the lower ranges are smaller than the (lower range) baseline value. Yet the same is not observed in Irish estimates, where the lower range estimate from the plausible and scenario limits exceeds the lower range baseline value. This difference stems from the differences in the citizen (TB/citizen) and employee (TB/employee) intensities between Germany and the Netherlands, compared to Ireland. Germany and the Netherlands have a larger difference in total data volume between aggregate citizen and employee use. In contrast, for Ireland, this difference is smaller, and therefore, the discount for employee use relative to citizen use has a higher share of fluctuation in the estimates.

Thus far, the uncertainties from D1 assume a purely domestic operation scenario, i.e., all cloud flow types have the same operating conditions as if they originated domestically. Nonetheless, imported cloud is prone to uncertainties surrounding the operating conditions (renewable mix) of the country of origin where the cloud data is hosted. In a real-life scenario, this should be expected to raise significant planning impacts and questions, i.e., does the decision to import cloud services from a country with a more renewable energy mix have a significant influence on the country's carbon emissions related to cloud data use in 2022?

Considering the uncertainties about tracing the data flow, the problem of import origin could not be addressed in the main text. To accurately estimate the impacts of imported cloud, it would be necessary to have a confident estimate of the origins of the data (e.g., 20% of imported cloud data in the Netherlands is initially hosted in Germany) – which is not available for any of the three cases. To mitigate uncertainties about the data flow, this work adopted the counterfactual scenario in the main text, in which the imported cloud has operating conditions (i.e., energy and carbon intensities) similar to those of the domestic conditions.

Notwithstanding, the counterfactual assumption has limitations in real-life cases. This part evaluates how differences in operating conditions for the share of imported cloud affect overall electricity consumption and corresponding emissions. This will take Ireland in 2022 as a reference case to showcase the influence. Considering uncertainties about the imported cloud's origin, this work considered five possible different geographical areas as the import source[118,119]:

- Area (a): EU member states, Norway, the UK, and Iceland

- Area (b): Balkans and Türkiye (Albania, Bosnia, Montenegro, North Macedonia, Serbia, Türkiye, Kosovo)
- Area (c): Eastern Europe and South Caucasus (Armenia, Azerbaijan, Georgia, Moldova, Ukraine)
- Area (d): Middle East and North Africa (Algeria, Egypt, Israel, Jordan, Lebanon, Morocco, Tunisia)
- Area (e): Central Asia (Kazakhstan, Kyrgyzstan, Tajikistan, Turkmenistan, Uzbekistan)

For each area, the emissions of imported cloud were calculated according to a 'regional emission factor: the regional average for the electricity consumption emission factor (t. CO_2e/MWh) from the national grid of all countries from the group[118,119]. To exemplify, the average regional factor for electricity consumption of group (a) in 2022 is estimated to be 0.286 t. CO_2e/MWh, which is the average of the electricity consumption emission factor from all EU member states, Iceland, Norway, and the UK for that year. The calculations used Eurostat data on the Covenant of Mayors for each region[118,119].

This estimate serves as the baseline for the electricity consumption of the imported cloud. Accurately calculating electricity consumption under domestic conditions across regions would require estimating each region's energy intensity (kWh/TB). However, the energy intensity method could not be used to evaluate this part because it requires historical data on annual data volume, which most countries do not report. The sensitivity analysis for Ireland is shown below:

Table S6. Variations on Cloud Emissions Estimations for Ireland in 2022 According to Origin of Imported Cloud (Region Average for Electricity Consumption)

	Upper Range				Lower Range			
	% Range from Baseline Emission Value		Total Cloud Emissions per Scenario According to Baseline Value (kt. CO_2eq)		% Range from Baseline Emission Value		Total Cloud Emissions per Scenario According to Baseline Value (kt. CO_2eq)	
	PS	SL	PS	SL	PS	SL	PS	SL
(A) EU, Iceland, Norway, UK	-3%	-3%	430	430	-3%	-3%	260	260
(B): Balkans and Türkiye	0	-2%	440	435	-1%	-1%	267	267
(C): Eastern Europe and South Caucasus	-1%	-2%	439	435	-1%	-1%	267	267
(D): Middle East and North Africa	-1%	-1%	439	439	-1%	-2%	267	265
(E): Central Asia	-1%	-2%	439	435	-1%	-1%	267	267

The table lays out the variations in results by region for Ireland. The Irish grid had a higher-than-average carbon intensity for electricity consumption when compared to the EU, Iceland, Norway, UK, and Eastern European grid averages, approaching similar levels of carbon intensity from the Balkans and Middle East for electricity consumption. However, given the relatively low share of cloud imports over the cloud total volume (~6%) and the relatively high carbon intensity of electricity consumption in Ireland, the impacts of foreign origins in Irish emission estimates for cloud computing are relatively minor and downwards, i.e., decreasing the emission amount for the same volume of imported data due to lower carbon intensity in other areas. Notwithstanding, geographical placement can

start to gain relevance for estimations when taking into consideration a data center hub where imports occupy a larger share from total of the cloud services.

Reference List

56. Hintemann, R., Hinterholzer, S. & Seibel, H. (2023). Rechenzentren in Deutschland: Aktuelle Marktentwicklungen – Update 2023. Borderstep Institute. https://www.bitkom.org/Bitkom/Publikationen/Studie-Rechenzentren-in-Deutschland.
57. Federal Network Agency (2022). Jahresbericht. German Federal Government. https://www.bundesnetzagentur.de/SharedDocs/Downloads/EN/BNetzA/PressSection/ReportsPublications/2023/AR2022.pdf.
58. Central Bureau of Statistics (2022). Elektriciteit geleverd aan datacenters, 2017-2021 dataset. Government of the Netherlands. https://www.cbs.nl/nl-nl/maatwerk/2022/49/elektriciteit-geleverd-aan-datacenters-2017-2021.
59. Central Statistics Office (2022). MEC02 Data Centres Metered Electricity Consumption. Government of Ireland. https://data.cso.ie/table/MEC02.
60. Deutsche Telekom (2017). The 2017 Financial Year.
61. Deutsche Telekom (2018). Corporate Social Responsibility Report 2018.https://report.telekom.com/annual-report-2018/.
62. Deutsche Telekom (2018). Aspect 1: Environmental concerns - Deutsche Telekom AG Annual Report 2018. (2018).https://report.telekom.com/annual-report-2018/management-report/corporate-responsibility-and-non-financial-statement/aspect-1-environmental-concerns.html.
63. Deutsche Telekom (2019). The 2019 Financial Year. https://report.telekom.com/annual-report-2019/
64. Deutsche Telekom (2019). Aspect 1: Environmental concerns - Deutsche Telekom AG Annual Report 2019. https://report.telekom.com/annual-report-2019/.
65. Deutsche Telekom (2020). The 2020 Financial Year. https://report.telekom.com/annual-report-2020/.
66. Deutsche Telekom (2021). The 2021 Financial Year. https://report.telekom.com/annual-report-2021/.
67. Deutsche Telekom (2022). Corporate Social Responsibility Report 2022. https://www.cr-report.telekom.com/2022/.
68. Deutsche Telekom (2023). Corporate Social Responsibility Report 2023. https://www.cr-report.telekom.com/2023/management-facts/environment/co2e-emissions.
69. KPN (2019). Appendix 7: Environmental Figures. https://jaarverslag2019.kpn/downloads/Environmental-figures.pdf.
70. KPN (2019). KPN Integrated Annual Report 2020. https://annualreport2019.kpn/.
71. KPN (2020). Appendix 11: Environmental Figures. https://www.jaarverslag2020.kpn/downloads/Environmental-figures.pdf.
72. KPN (2020). KPN Integrated Annual Report 2020. https://ir.kpn.com/files/shareholder_meetings/2021/Integrated_Annual_Report_2020_AGM_2021.pdf.

73. KPN (2021). KPN Integrated Annual Report 2021. https://ir.kpn.com/files/doc_financials/2021/ar/KPN_Integrated_Annual_Report_2021.pdf.
74. KPN (2021). Appendix 6: Environmental Figures. https://www.jaarverslag2021.kpn/downloads/Environmental-figures.pdf.
75. KPN (2022). KPN Integrated Annual Report 2022. https://ir.kpn.com/files/doc_financials/2022/ar/KPN_Integrated_Annual_Report_2022_20230224.pdf.
76. KPN (2023). KPN Integrated Annual Report 2023. https://ir.kpn.com/files/doc_financials/2023/ar/KPN-Integrated-Annual-Report-2023.pdf.
77. Virgin Media (2019). Our carbon footprint in the UK and Ireland 2014-2019. https://virginmedia.com/sustainability.
78. Virgin Media (2020). Our carbon footprint in the UK and Ireland 2014-2020. https://virginmedia.com/sustainability.
79. Virgin Media O2 (2022). Sustainability Performance Update 2022. https://news.virginmediao2.co.uk/wp-content/uploads/2023/06/Sustainability-Performance-Update-2022.pdf.
80. Virgin Media O2 (2023). Sustainability Performance Update 2023. https://news.virginmediao2.co.uk/wp-content/uploads/2024/04/Sustainability-Performance-Update-2023-1.pdf.
81. Environmental Agency (2023). Entwicklung der spezifischen Treibhausgas-Emissionen des deutschen Strommix in den Jahren 1990 – 2022 dataset. German Federal Government. https://www.umweltbundesamt.de/sites/default/files/medien/1410/publikationen/2023_05_23_climate_change_20-2023_strommix_bf.pdf.
82. Central Bureau of Statistics (2023). CO2 emissiefactor, fossiel energieverbruik en rendement voor elektriciteit afgeleverd bij elektriciteitsverbruiker dataset. Government of the Netherlands https://www.cbs.nl/nl-nl/maatwerk/2023/51/rendementen-co2-emissie-elektriciteitsproductie-2022.
83. Sustainable Energy Authority of Ireland (SEAI) (2024). CO_2 Emissions dataset. Government of Ireland . https://www.seai.ie/data-and-insights/seai-statistics/key-statistics/co2/.
84. Environmental Agency (2024). Emission of greenhouse gases covered by the UN Framework Convention on Climate dataset. German Federal Government. https://www.umweltbundesamt.de/daten/klima/treibhausgas-emissionen-in-deutschland#emissionsentwicklung.
85. Central Bureau of Statistics (2023). Emissies van broeikasgassen berekend volgens IPCC-voorschriften dataset. Government of the Netherlands. https://www.cbs.nl/nl-nl/cijfers/detail/70946ned.
86. Sustainable Energy Authority of Ireland (SEAI) (2024). CO_2 Emissions dataset. Government of Ireland. https://www.seai.ie/data-and-insights/seai-statistics/key-statistics/co2/.
87. Bundesnetzagentur (2019). Annual Report 2019. German Federal Government. https://data.bundesnetzagentur.de/Bundesnetzagentur/SharedDocs/Downloads/EN/BNetzA/PressSection/ReportsPublications/2020/annualreport19.pdf.

88. Bundesnetzagentur (2020). Annual Report 2020. German Federal Government. https://data.bundesnetzagentur.de/Bundesnetzagentur/SharedDocs/Downloads/EN/BNetzA/PressSection/ReportsPublications/2021/annualreport20.pdf.
89. Bundesnetzagentur (2021). Annual Report 2021. German Federal Government. https://data.bundesnetzagentur.de/Bundesnetzagentur/SharedDocs/Downloads/EN/BNetzA/PressSection/ReportsPublications/2022/ar2021.pdf.
90. Bundesnetzagentur (2022). Annual Report 2022. German Federal Government. https://www.bundesnetzagentur.de/SharedDocs/Downloads/EN/BNetzA/PressSection/ReportsPublications/2023/AR2022.pdf?__blob=publicationFile&v=1.
91. VATM & Dialog Consult (2020). 22nd Telecommunications Market Analysis Germany 2022. https://www.vatm.de/wp-content/uploads/2020/10/VATM_TK-Marktstudie-2020_1020_a.pdf.
92. VATM & Dialog Consult (2021). 23rd Telecommunications Market Analysis Germany 2022. https://www.vatm.de/wp-content/uploads/2021/10/VATM_TK-Marktstudie_281021_f.pdf .
93. VATM & Dialog Consult (2022). 24th Telecommunications Market Analysis Germany 2022. https://www.vatm.de/wp-content/uploads/2022/10/TK-Marktstudie-2022_DC-VATM_261022.pdf.
94. VATM & Dialog Consult (2023). 25th Telecommunications Market Analysis Germany 2023. https://www.vatm.de/wp-content/uploads/2023/11/Marktstudie-2023-V6.pdf.
95. Autoriteit Consument & Markt (ACM) (2024). Telecommonitor dataset. https://public.tableau.com/app/profile/autoriteit.consument.en.markt/viz/Telecommonitor/OVER.
96. TU Eindhoven & Dialogic (2016). Beyond Fast: How the Speed of Residential Internet Access Will Develop Between Now and 2022. https://rbekkers.ieis.tue.nl/Dialogic_2016.16_%20Beyond_fast.pdf.
97. van der Vorst, T. (2018). Understanding the Demand Growth for Digital Connectivity. Dialogic. https://dialogic.nl/wp-content/uploads/2018/01/20180619-Tommy-van-der-Vorst-Understanding-the-demand-for-bandwidth-presentation-for-UFBB_public.pdf.
98. Commission for Communications Regulation (CommReg) (2024). Internet Statistics dataset. https://www.comreg.ie/industry/electronic-communications/data-portal/downloads/.
99. Destatis Statistiches Bundesamt (2024). Public Service Personnel: Germany dataset. https://www-genesis.destatis.de/genesis/online?operation=abruftabelleBearbeiten&levelindex=1&levelid=1715086504269&auswahloperation=abruftabelleAuspraegungAuswaehlen&auswahlverzeichnis=ordnungsstruktur&auswahlziel=werteabruf&code=74111-0001&auswahltext=&werteabruf=Value+retrieval#abreadcrumb.
100. Eurostat (2023). Employment A*10 Industry Breakdowns dataset. https://data.europa.eu/data/datasets/miiqtkdxfswgkzqp7kcig?locale=en.
101. Eurostat (2021). Individuals - Use of Cloud Services dataset. https://doi.org/10.2908/ISOC_CICCI_USE.
102. Cisco (2023). VNI Complete Forecast Highlights. https://www.cisco.com/c/dam/m/en_us/solutions/service-provider/vni-forecast-highlights/pdf/Global_Business_Highlights.pdf .
103. Borderstep Institute & Deutsche Bundesbank (2022). Foreign Trade ICT Services 2019-2021 dataset. Bitkom e.V. https://www.bitkom.org/Marktdaten/ITK-Konjunktur/index.jsp.

104. Borderstep Institute & Deutsche Bundesbank (2024). Foreign Trade ICT Services 2022-2023 dataset. Bitkom e.V. https://www.bitkom.org/Marktdaten/ITK-Konjunktur/index.jsp.

105. Centraal Bureau voor de Statistiek (CBS) (2022). International Trade; Imports and Exports of Services 2014-2020 dataset. https://opendata.cbs.nl/#/CBS/en/dataset/82616ENG/table.

106. Centraal Bureau voor de Statistiek (CBS)(2024). International Trade; Imports and Exports of Services by Country, Quarter dataset. https://opendata.cbs.nl/#/CBS/en/dataset/84765ENG/table.

107. Central Statistics Office (CSO) (2024). BPA03 Exports and Imports of Services dataset. https://ws.cso.ie/public/api.restful/PxStat.Data.Cube_API.ReadDataset/BPA03/XLSX/2007/en.

108. Büro für Technikfolgen-Abschätzung beim Deutschen Bundestag (TAB) (2022). Energy Consumption of ICT Infrastructure. TAB Working Report No. 198. https://www.tab-beim-bundestag.de/projekte_energieverbrauch-der-ikt-infrastruktur.php.

109. International Telecommunications Union (ITU) (2018). L.1450 Methodologies for the Assessment of the Environmental Impact of the Information and Communications Technology Sector.

110. World Resources Institute (2011). Greenhouse Gas Protocol – Scope 3 Standard. https://ghgprotocol.org/sites/default/files/standards/Corporate-Value-Chain-Accounting-Reporing-Standard_041613_2.pdf

111. World Resources Institute (2017). ICT Sector Guidance built on the GHG Protocol Product Life Cycle Accounting and Reporting Standard. https://ictfootprint.eu/en/ghg-protocol-hardware-factsheet.

112. International Energy Agency (IEA) (2024). Analysis and Forecast to 2026 https://iea.blob.core.windows.net/assets/6b2fd954-2017-408e-bf08-952fdd62118a/Electricity2024-Analysisandforecastto2026.pdf.

113. Foley, P., Dog, F., Gemmell, A., Rys, J., & Vinciguerra, C. (2023). Economic Value of Data Flows: Final Study Report. European Commission - Directorate-General for Communications Networks, Content and Technology. https://digital-strategy.ec.europa.eu/en/library/economic-value-data-flows.

114. Grant Thorton (2018). A Study of the Economic Benefits of Data Centre Investment in Ireland. https://www.idaireland.com/getmedia/98975918-818a-499d-b4e5-6edc885d8412/IDA-Ireland-Economic-Benefits-of-Data-Centre-Investment-Final-May182018.pdf.

115. McDermott, K. (2023). The Gateway to the EU: Why Dublin Became a Bustling Tech Hub. Euronews https://www.euronews.com/next/2023/02/02/the-gateway-to-the-eu-why-dublin-became-a-bustling-tech-hub.

116. Thales Group (2022). 2022 Thales Data Threat Report: Navigating Data Security in an Era of Hybrid Work, Ransomware and Accelerated Cloud Transformation. https://cpl.thalesgroup.com.

117. European Commission (2023). First Report on the State of the Digital Decade Calls for Collective Action to Shape the Digital Transition. https://ec.europa.eu/commission/presscorner/detail/en/ip_23_4619.

118. Bastos, J., Monforti-Ferrario, F., Melica, G. (2024). GHG Emission Factors for Electricity Consumption. European Commission, Joint Research Centre (JRC) dataset. http://data.europa.eu/89h/919df040-0252-4e4e-ad82-c054896e1641.

119. Bastos, J., Monforti-Ferrario, F., Melica, G. (2024). Covenant of Mayors for Climate and Energy: greenhouse gas emission factors for local emission inventories, Covenant of Mayors collection - 2024 datasets, Publications Office of the European Union. 10.2760/014585.
